# *E. Coli* cytotoxic necrotizing factor-1 promotes colorectal carcinogenesis by causing oxidative stress, DNA damage and intestinal permeability alteration

**DOI:** 10.1186/s13046-024-03271-w

**Published:** 2025-01-29

**Authors:** Michela Tozzi, Alessia Fiore, Sara Travaglione, Francesca Marcon, Gabriella Rainaldi, Elena Angela Pia Germinario, Ilenia Laterza, Simona Donati, Daniele Macchia, Massimo Spada, Omar Leoni, Maria Cristina Quattrini, Donatella Pietraforte, Sofia Tomasoni, Filippo Torrigiani, Ranieri Verin, Paola Matarrese, Lucrezia Gambardella, Francesca Spadaro, Maria Carollo, Agostina Pietrantoni, Francesca Carlini, Concetta Panebianco, Valerio Pazienza, Filomena Colella, Donatella Lucchetti, Alessandro Sgambato, Antonella Sistigu, Federica Moschella, Marco Guidotti, Olimpia Vincentini, Zaira Maroccia, Mauro Biffoni, Roberta De Angelis, Laura Bracci, Alessia Fabbri

**Affiliations:** 1 Department of Oncology and Molecular Medicine, Istituto Superiore di Sanità, Rome, Italy; 2 Department of Cardiovascular, Endocrine-Metabolic Diseases and Aging, Istituto Superiore di Sanità, Rome, Italy; 3 Department of Environment and Health, Istituto Superiore di Sanità, Rome, Italy; 4 Center of Animal Research and Welfare, Istituto Superiore di Sanità, Rome, Italy; 5 Core Facilities, Istituto Superiore di Sanità, Rome, Italy; 6 Department of Comparative Biomedicine and Food Science, BCA-University of Padua, Legnaro, PD Italy; 7 Center for Gender-Specific Medicine, Istituto Superiore di Sanità, Rome, Italy; 8 Division of Gastroenterology, Fondazione IRCCS “Casa Sollievo della Sofferenza”, San Giovanni Rotondo, FG Italy; 9 Multiplex Spatial Profiling Center, Fondazione Policlinico Universitario “A. Gemelli” – IRCCS, Rome, Italy; 10 Department of Translational Medicine and Surgery, Università Cattolica del Sacro Cuore, Rome, Italy; 11 Fondazione Policlinico Universitario “A. Gemelli” – IRCCS, Rome, Italy; 12 Department of Food Safety, Nutrition and Veterinary Public Health, Istituto Superiore di Sanità, Rome, Italy

**Keywords:** Colorectal cancer, CNF1, Escherichia coli, Oxidative stress, DNA damage, Genotoxicity, Inflammation, Intestinal permeability, 3D Caco-2 spheroids, Immune infiltrates

## Abstract

**Background:**

Bacterial toxins are emerging as promising hallmarks of colorectal cancer (CRC) pathogenesis. In particular, Cytotoxic Necrotizing Factor 1 (CNF1) from *E. coli* deserves special consideration due to the significantly higher prevalence of this toxin gene in CRC patients with respect to healthy subjects, and to the numerous tumor-promoting effects that have been ascribed to the toxin in vitro. Despite this evidence, a definitive causal link between CNF1 and CRC was missing. Here we investigated whether CNF1 plays an active role in CRC onset by analyzing pro-carcinogenic key effects specifically induced by the toxin in vitro and in vivo.

**Methods:**

Viability assays, confocal microscopy of γH2AX and 53BP1 molecules and cytogenetic analysis were carried out to assess CNF1-induced genotoxicity on non-neoplastic intestinal epithelial cells. Caco-2 monolayers and 3D Caco-2 spheroids were used to evaluate permeability alterations specifically induced by CNF1, either in the presence or in the absence of inflammation. In vivo, an inflammatory bowel disease (IBD) model was exploited to evaluate the carcinogenic potential of CNF1. Immunohistochemistry and immunofluorescence stainings of formalin-fixed paraffin-embedded (FFPE) colon tissue were carried out as well as fecal microbiota composition analysis by 16 S rRNA gene sequencing.

**Results:**

CNF1 induces the release of reactive oxidizing species and chromosomal instability in non-neoplastic intestinal epithelial cells. In addition, CNF1 modifies intestinal permeability by directly altering tight junctions’ distribution in 2D Caco-2 monolayers, and by hindering the differentiation of 3D Caco-2 spheroids with an irregular arrangement of these junctions. In vivo, repeated intrarectal administration of CNF1 induces the formation of dysplastic aberrant crypt foci (ACF), and produces the formation of colorectal adenomas in an IBD model. These effects are accompanied by the increased neutrophilic infiltration in colonic tissue, by a mixed pro-inflammatory and anti-inflammatory cytokine milieu, and by the pro-tumoral modulation of the fecal microbiota.

**Conclusions:**

Taken together, our results support the hypothesis that the CNF1 toxin from *E. coli* plays an active role in colorectal carcinogenesis. Altogether, these findings not only add new knowledge to the contribution of bacterial toxins to CRC, but also pave the way to the implementation of current screening programs and preventive strategies.

**Supplementary Information:**

The online version contains supplementary material available at 10.1186/s13046-024-03271-w.

## Graphical abstract

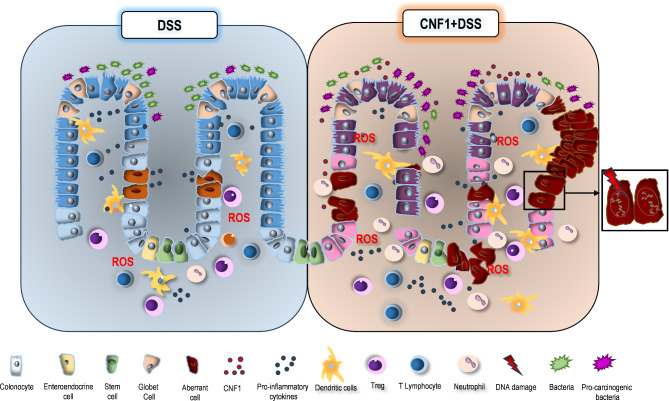

## Background

Colorectal cancer (CRC) is the 3rd most common type of cancer and the 2nd cause of cancer death worldwide [1]. More than 90% of CRC patients are diagnosed after 50 years of age, and for this reason CRC has been ordinarily considered a disease associated with aging [2]. Nevertheless, current data highlight a rise in CRC incidence among young individuals (18–49 years of age) living in high-income countries [3], emphasizing the strong impact of lifestyle and environmental factors on this cancer type. Mechanisms contributing to the early-onset of CRC remain elusive, especially in those young adults lacking a family history of CRC or polyps. Over the past two decades, research has shown that dysbiosis, i.e., a gut microbiota imbalance, could be a risk factor for CRC development and progression [4, 5]. Located in close proximity to the intestinal epithelium, the gut microbiota encompasses a large number of microorganisms that closely interact with epithelial cells and are involved in many physiological processes, including immunity and metabolism. It is well established that gut microbiota dysbiosis significantly contributes to inflammatory conditions [6], and that prolonged inflammation alters the tissue microenvironment, leading to tissue reshaping, immune suppression, DNA damage, and genomic instability, all factors contributing to carcinogenesis [7]. To deeply understand microbial involvement in the development of CRC, the “driver-passenger” carcinogenesis model has been proposed [8]. According to this model, the initiation of CRC is triggered by the local colonization of the gut mucosa by specific pathogens, which can function as “drivers” of cancer. These driver microorganisms cause changes in the tumor microenvironment, allowing for colonization by opportunistic (passengers) microorganisms that facilitate disease progression. Evidence exists that passenger bacteria can turn into driver bacteria when the process of tumorigenesis is accompanied by gut permeability alterations and damage, which changes the microenvironment and the microbial selective pressure [9]. Indeed, it has been demonstrated that infection with some bacteria leads to the occurrence of different types of gastro-intestinal cancers. This is the case of *Helicobacter pylori* infection associated with gastric cancer [10], and of *Campylobacter jejuni* and *Salmonella typhi* infection, associated with small intestinal lymphomas and hepatobiliary carcinoma respectively [11]. Recent literature testifies that not only specific microorganisms, but also their virulence factors or specific metabolites can induce human carcinogenesis [7]. It was reported that estrogen-metabolizing enzymes from gut-colonizing bacteria may contribute to hormone-dependent breast cancer [12]. It was also reported that toxins such as the cytolethal distending toxin (CDT) and the colibactin produced by *Escherichia coli*, as well as BFT from enterotoxigenic *Bacteroides fragilis* promote CRC [13, 14]. In this respect, other virulence factors from defined *E. coli* strains colonizing gut microbiota are emerging as potential players in CRC onset. Data stemming from a previous study indicate that the cycle inhibiting factor (CIF) toxin gene from *E. coli* is significantly associated with pre-cancerous lesions of colon-rectum, as compared to healthy tissue. In contrast, toxins from *E. coli* as a whole have a higher incidence in adenocarcinoma patients [15]. Among the *E. coli* toxins, the cytotoxic necrotizing factor-1 (CNF1) gene is overrepresented in CRC patients colonized by *E. coli* [16, 17]. CNF1 is a protein toxin that permanently activates Rho GTPases (Rho, Rac and Cdc42), molecular switches involved in regulating a wide range of cell signaling pathways. By activating Rho GTPases, CNF1 induces cytoskeletal and cell cycle alterations, with subsequent formation of multipolar metaphases [18] and megalocytic and multinucleated cells [19]. CNF1 also increases cellular motility [20], confers resistance to apoptotic stimuli [21, 22], and promotes quiescent cells entry into the cell cycle [23]. Besides exerting direct effects on epithelial cells, CNF1 can also modulate the interplay between epithelial and immune cells by stimulating the NFkB-dependent production of proinflammatory factors [24, 25], which, in turn, mobilize Gr1-positive myeloid cells [26]. Despite the epidemiological association and the numerous in vitro evidence supporting the role of CNF1 in colorectal carcinogenesis, a definitive causal link between CNF1 and CRC is still missing. In the present study, we demonstrate for the first time to our knowledge, that exposure of intestinal epithelial cells to CNF1 favors CRC development in vivo. We provide evidence of the genotoxic effect of this toxin and of the synergism between CNF1 and inflammatory signals in gut barrier disruption in vitro and in vivo. We propose that, by inducing DNA damage and the permeabilization of the intestinal mucosa, CNF1 can synergize with inflammatory bowel disease (IBD) in CRC development. This effect is paralleled by a CNF1-induced reshaping of the gut microbiota towards a CRC-permissive microenvironment and by the recruitment of neutrophils into the colonic mucosa. Our results support the possibility that CNF1 produced by *E. coli* is a novel risk factor for CRC, especially in the context of inflamed colon tissue, and deserves further clinical investigation to implement current screening programs.

## Methods

### Cell lines and reagents

IEC-6 cells (normal rat small intestine, ATCC CRL1592) were cultured at 37 °C with 5% CO_2_ in tissue culture flasks or Petri dishes with Dulbecco’s modified Eagle’s Medium (DMEM) + GlutaMax (Gibco) supplemented with 10% heat-inactivated fetal bovine serum (FBS; Euroclone), 10 µg/ml of insulin (Sigma-Aldrich), 100 U/ml penicillin, and 100 µg/ml streptomycin. The human monocytic THP-1 cell line (ATCC TIB-202) was maintained in RPMI 1640 containing 10% heat-inactivated FBS, 100 U/ml penicillin, 100 µg/ml streptomycin and 2 mM L-glutamine. The human Caco-2 cell line (ATCC-HTB-37) was cultured with DMEM high glucose, 10% heat- inactivated FBS, 100 U/ml penicillin, and 100 µg/ml streptomycin, 2 mM L-glutamine and 1% non-essential amino acids (NEAA). Human primary colonic epithelial cells (HPCECs; Cell Biologics), isolated from normal human colon tissue, were maintained in tissue culture flasks or Petri dishes pre-coated with gelatin-based coating solution (Cell Biologics) and cultured with Complete Human Epithelial Cell Medium/w Kit (Cell Biologics) supplemented with 10% heat-inactivated FBS. Cultured cells were used up to passage 15. All cell lines were routinely tested to confirm the absence of mycoplasma contamination. CNF1 and CNF1 C866S were purified as previously described [27]. All preparations tested were negative for LPS contamination (Pierce, Chromogenic Endotoxin Quant Kit).

### Three-dimensional Caco-2 spheroids

To obtain 3D spheroids, Caco-2 cells were cultured in presence of Matrigel (Matrix Basement Membrane, Corning). Specifically, Matrigel was added onto 12 mm sterile glass slips placed inside 24-well tissue culture plates, and allow to solidify at 37 °C for 30 min. Caco-2 cell monolayer was detached from the substratum by using 0.02% EDTA and 0.25% trypsin solutions, and resuspended in a medium consisting of DMEM high glucose (supplemented with 100 U/ml penicillin,100 µg/ml streptomycin, 2 mM L-glutamine and 1% NEAA) and DMEM low glucose (1.0 g/L glucose; supplemented with 100 U/ml penicillin and 100 µg/ml streptomycin and 2 mM L-glutamine), both FBS-free, and mixed together at ratio 1:1 (v/v), and added with 40% Matrigel (v/v). Cell suspension (5,000 cells/well) was plated onto solidified Matrigel and, after incubation at 37˚C in a 5% CO_2_ atmosphere, DMEM 2.75 g/L glucose, supplemented with 5% heat-inactivated FBS, 1% glutamine, 0.5% non-essential amino acids, and 1% penicillin and streptomycin, was added to each well. All samples were incubated at 37˚C in a 5% CO_2_ atmosphere. After 2 days of growth, cultures were treated with 1.5 pM CNF1. At 9 days of culture, spheroids samples were analysed according the experiments described below.

### MTT assay, viable cell count and apoptosis

IEC-6 and HPCEC cells were plated in flat-bottomed 96-well plates in quintuplicate (500 cells/well in 0.1 ml of complete medium) and placed in the incubator at 37˚C with 5% CO_2_. After 24 h, 0.1 ml of medium containing CNF1 or CNF1-C866S (25 − 1 pM final concentration) were added to each well and plates were incubated for up to 6 days. At the time of the assay, 0.02 ml of MTT [3-(4.5-dimethyl-thiazol-2-y1) 2.5-diphenyl tetrazolium bromide, Sigma-Aldrich, 5 mg/ml in sterile PBS] were added to each well for 3 h and the assay was completed according to the manufacturer’s instructions. The absorbance was read at 570 nm by using a MultiSkan FC microplate (Thermo Fisher Scientific) and results were expressed as the mean ± SD percentage of viability compared to untreated cells (negative control considered as 100% of viability). For viable cell count, 15,000 cells/well were cultured in triplicate in flat-bottomed 6-well plates for 24 h before the addition of CNF1 or CNF1-C866S (25 or 10 pM). After 3 and 6 days of culture, cells were trypsinized, diluted in trypan blue solution (0.4% in PBS) and counted by using a Neubauer counting chamber. For apoptosis detection, cell suspensions were stained using the Dead Cell Apoptosis Kit (Invitrogen, Thermo Fisher Scientific) according to the manufacturer’s instructions and immediately acquired on a Gallios flow cytometer (Beckman Coulter) and analysed with Kaluza software (Beckman Coulter). At least 20,000 events per sample were acquired.

### Cell cycle analysis

IEC-6 cells (10^5^ cells) were seeded, in duplicate, in 90 mm Petri dishes and cultured for 24 h before the addition of 25 pM CNF1. After 16 h of culture, control and treated IEC-6 cells were trypsinized and harvested in PBS, followed by fixation in ice-cold 70% ethanol overnight at 4 °C. 3 × 10^5^ cells were stained with a mixture of propidium iodide (50 µg/ml, Sigma-Aldrich) and RNAse 1-A (0.2 mg/mL) for 30 min at room temperature (RT) in the dark. Samples were analysed by using a FACSCalibur flow cytometer (BD Biosciences) equipped with a 488 nm Argon laser and with a 635 red diode laser (BD Biosciences). The PI-stained cells were analysed by collecting FL2 red fluorescence in a linear scale at above 620 nm. The percentage of cells in the different phases of the cell cycle was determined by ModFIT software analysis (Becton Dickinson). Apoptotic cells and debris were excluded and at least 20,000 events per sample were acquired. Each experiment was performed at least 3 times.

### Fluorescence and confocal laser scanning microscopy (CLSM) analysis

IEC-6 and HPCEC (15,000 cells/well) cells were seeded on glass coverslips in 24-well plates. After 24 h, 25 pM CNF1 or CNF1-C8SS6 were added for different time points (1-3-5-7-24 h). For N-acetyl cysteine (NAC) inhibition experiments IEC-6 cells were pre-treated with 10 M NAC for 2 h before CNF1 addition for 24 h. Cells were fixed in 4% paraformaldehyde (PFA) for 10 min at RT, followed by two washes with PBS, and permeabilization with 0.4% Triton X-100 in PBS for 10 min at RT. The cells were incubated with PBS with 3% BSA for 40 min at RT and thereafter the primary antibodies were added for 1 h at 37 °C while the secondary antibodies were added for 1 h at RT (see Supplementary Table 1 for details). Finally, the cells were incubated for 5 min with Hoechst 33,258 (Invitrogen) for nuclei labelling. For F-actin staining, cells were incubated with TRITC-phalloidin (See Supplementary Table 1) for 40 min at RT. For F-actin and pATR staining cells were observed with an optical microscope Olympus BX51/BX52, and images were acquired using the program IAS 2000 (Delta System). For histone H2AX-Ser139 (γH2AX) and P53-binding protein 1 (53BP1), CLSM observations were performed with a Zeiss LSM980 apparatus, using a 63x/1.40 NA oil objectives and excitation spectral laser lines at 405, 488 and 594 nm. Image acquisition and processing were carried out using the Zeiss confocal software Zen 3.3 (Blue edition) and Adobe Photoshop CS5 software programs (Adobe Systems). Signals from different fluorescent probes were taken in sequential scan settings. For γH2AX and 53BP1 foci count, 100 nuclei were counted and cells were classified into the following classes: 0–5, 5–10, 10–20 or > 20 of foci number per nucleus.

For CLSM analyses on Caco-2 monolayers and 3D spheroids cells were fixed with 3% PFA for 30 min at 4 °C and permeabilized with 0.5% Triton X-100 for 10 min at RT. Primary monoclonal anti-ZO-1 antibody (Becton Dickinson) was incubated for 90 min at RT in 0,5% Triton-X-100/3% BSA/3%FCS, followed by 1 h incubation at RT with Alexa Fluor-488 F(ab)2 fragments of goat anti-mouse IgG and Alexa Fluor-594 phalloidin (Thermo Fisher Scientific). Nuclei were stained with 4’,6-diamidino-2-phenylindole (DAPI) (Thermo Fisher Scientific). At the end of the staining, membranes of Caco-2 monolayers were finally cut from the scaffold, placed on glass slides and coverslips were mounted with Vectashield mounting medium for fluorescence (Vector Laboratories) and observed with a Zeiss LSM980 as above described using a 20x/0.8 NA objective for spheroids and a 40x/1.40 NA oil objective for Caco-2 monolayers. The mean fluorescence intensity (MFI) of ZO-1 in Caco-2 monolayers after the different cell treatments was calculated by ImageJ software.

### Western blot analysis

IEC-6 and HPCEC cells (10^6^) were seeded, in triplicate, in 90 mm Petri dishes and cultured for 24 h before the addition of 25 pM CNF1 or CNF1-C866S. At the indicated time-points, cells were lysed in cold RIPA buffer and protein extracts were subjected to Western blot (WB) analyses as previously described [28]. Detailed information on the antibodies, including clone and dilution is provided in Supplementary Table 1.

### ROS production

IEC-6 cells (10^6^) were cultured for 24 h in a 90 mm Petri dish and subsequently incubated for 4, 7–24 h with CNF1 (25 pM). The production of reactive oxidizing species (ROS) was established by the oxidation of the probe 1-hydroxy-3-carboxypyrrolidine (CPH; ENZO Life Sciences), to the stable 3-carboxy-proxyl radical (CP^●^), which can be detected by electron paramagnetic resonance spectroscopy (EPR) by using a EPR Bruker ECS106 (Bruker Italia). CP^●^ formation is not specific to a single oxidant species but is useful for monitoring all ROS (e.g. superoxide anion, hydroxyl radical, peroxynitrite, etc.) formed in the biological system under study. 0.1 ml of cell suspension was incubated with 0.002 ml of CPH 50 mM (final concentration 0.5 mM) for 10 min at 37 °C in air. Samples were loaded into a gas-permeable Teflon tube with an inner diameter of 0.81 mm and wall thickness of 0.05 mm (Zeus Industrial Products). The Teflon tube was bent twice, inserted into a quartz tube, and attached to the cavity (4108 TMH) of EPR. Spectra were acquired exactly 12 min after CPH addition to cell suspensions and ROS formation was expressed as amount of CP^●^/mg protein (protein measured by the bicinchonic acid method).

### Analysis of anaphase cells

IEC-6 cells (1.5 × 10^5^) were seeded in 60 mm Petri dishes containing a sterile coverslip 22 × 22 mm, and cultured for 24 h before treatment. Twenty-four hours after incubation with CNF1 (25 pM), cells were gently washed with 0.9% NaCl at 4 °C, incubated at 4 °C in cold 1% sodium citrate for 20 min, fixed 3 times with cold methanol/acetic acid (3:1 ratio; 10 min each step at 4 °C), and finally stained for 10 min with 5% Giemsa (Carlo Erba) in Sorensen Buffer (Na_2_HPO_4_–KH_2_PO_4_, pH 6.8). One hundred anaphases for each experimental point were examined for the analysis of chromosome displacement, chromosome bridges and anaphase morphology. Two independent experiments were carried out.

### Analysis of chromosome aberrations

IEC-6 cells (1.5 × 10^5^) were seeded in 60 mm Petri dishes and treated for 24 h with CNF1 (25 pM). Two hours before harvesting, Colcemid (0.1 ug/ml) was added to the cultures to allow the accumulation of cells in metaphase. Cells were then trypsinized and incubated for 10 min at 37 °C in 0.075 M KCl before fixing with cold methanol/acetic acid (3:1) solution. Slides were made by a conventional air-drying technique and stained with 5% Giemsa solution (Carlo Erba) in Sorensen Buffer (Na_2_HPO_4_–KH_2_PO_4_, pH 6.8) for 10 min at RT. To evaluate the persistence of DNA damage in subsequent cell divisions, 30 µM 5-bromodeoxyuridine (BrdU; Sigma-Aldrich) was added to IEC-6 cultures for 24 and 48 h after the removal of CNF1. Cells were then harvested as described above. To identify metaphases that have undergone two or more cell cycles after treatment, the classical staining to differentiate sister chromatids [29] was applied with minor modifications. Slides were incubated in 250 µg/ml Hoechst solution (Hoechst 33258, Sigma-Aldrich) for 30 min at RT and then exposed for 20 min to light from an Osram Ultra-Vitalux sunlamp, in coplin jars containing 0.2 × SSC. The slides were stained with 5% Giemsa solution (Carlo Erba) in Sorensen Buffer (Na_2_HPO_4_–KH_2_PO_4_, pH 6.8) for 10 min. Two hundred metaphases were analyzed for each experimental condition.

### Trans-epithelial electrical resistance (TEER) monitoring

Caco-2 cells were seeded (65 × 10^3^) on cell culture inserts (24-Well Insert 3.0 μm PET translucent, cellQART) placed in 24-well plates. Cells were allowed to grow for 21 days with 0.3 ml of culture medium in the insert and 1 ml of culture medium in the well. The medium was replaced 2–3 times a week. On day 21 of culture, TEER was recorded by using (Electrical Resistance System; Merck-Millipore). TEER values between 300 and 400 Ω x cm^2^ were considered acceptable to proceed with the specific treatments. TEER measurements were then repeated 6, 24 and 48 h after treatment of Caco-2 monolayers with 25 pM CNF1 or 25 pM CNF1-C866S or CNF1 + inflammatory supernatant (diluted 1:15 in culture medium) generated by stimulation of THP1 cells according to a previously published protocol [30]. Three-to-six TEER measurements were recorded for each insert at each time-point.

### Scanning electron microscopy (SEM) and transmission electron microscopy (TEM) analysis

To isolate Caco-2 spheroids from Matrigel, samples were incubated with a cell recovery solution (ratio 1:2; CORNING, Thermo Fisher Scientific) for 45 min at 4 °C and centrifuged at 76 x *g* for 5 min.

For SEM observation, samples were first fixed with 2.5% glutaraldehyde in Na-cacodylate buffer 0.1 M, post-fixed with 1% OsO_4_, dehydrated through a graded series of ethanol and hexamethyldisilazane solutions, dried and left to evaporate for 2 h. Dried samples were mounted on stubs, gold coated and imaged using secondary electrons with QUANTA INSPECT F (FEI) microscope.

For TEM analysis, samples were prepared according to a previously published protocol [31] with minor changes. Briefly, Caco-2 spheroids were fixed with glutaraldehyde 2.5%, PFA 4%, CaCl_2_ 2 mM in Na-cacodylate buffer 0.1 M overnight at 4 °C, post-fixed with 2% OsO_4_ in cacodylate buffer for 1 h. Samples were then treated with 1% Tannic Acid for 30 min at RT followed by incubation with 20% ethanol for 10 min and UA-Zero EM Stain (Agar Scientific) for 1 h. Spheroids were dehydrated by serial concentrations of alcohol solutions, incubated with propylene oxide and embedded in Agar 100 (Agar Scientific). Ultrathin sections, obtained by UC6 ultramicrotome (Leica Microsystems), were examined at 100 kV with FEI/Philips EM 208 S TEM equipped with acquisition system/Megaview SIS camera (Olympus).

### Colorectal carcinogenesis model

Eight-to-nine-week-old female C57BL/6 mice (ENVIGO RMS) were housed in the Animal Facility of the Istituto Superiore di Sanità. Food and water were provided *ad libitum*, unless otherwise specified. The mice were randomly divided into two experimental groups [CNF1 and CNF1 + dextran sulfate sodium (DSS)] and three control groups [PBS, DSS and azoxymethane (AOM) + DSS]. Experimental colitis was induced by the repeated administration of DSS (2% w/v; MW: 36,000–50,000 kDa; MP Biomedicals) in the drinking water for 7 days followed by 14 days of autoclaved tap water. Some animals were anesthetized (100 mg/Kg Ketamine + 10 mg/Kg Xylazine) and received the intrarectal (i.r.) administration of CNF1 (25 pM) in 0.05 ml of PBS at the beginning of each DSS cycle. Another group of mice received two intraperitoneal injections of AOM (10 mg/kg) 7 days apart before the first DSS administration to induce colitis-associated colorectal (CAC) tumors as previously described [32]. To follow the onset of tumors, anaesthetized mice underwent colonoscopies with the Coloview mini-endoscopic system (Karl Storz) according to a previously published protocol [33]. The severity of the disease was scored according to the parameters reported in [33]. Fecal pellets were collected from all groups three and six months after treatment and stored at -80 °C until use. To evaluate intestinal permeability, mice were fasted for 4 h before being gavaged with 0.15 ml of PBS containing 80 mg/ml 4 kDa FITC-dextran (Sigma-Aldrich). After 4 h, blood was collected from the submandibular vein and placed in K3-EDTA-coated tubes (Sarsted). Each tube was gently mixed by inversion and centrifuged at 1500 x *g* for 10 min at 4 °C. Plasma was transferred into a new Eppendorf 1.5 ml tube and diluted to 1:5 and 1:10 in sterile PBS. 0.1 ml of each dilution was then transferred to 96-well plates in triplicate and FITC-dextran concentration was measured by using a spectrophotometer at 530 nm.

### Histopathology

Three portions of large intestine from each animal were collected and fixed in formalin for 48 h and were routinely processed for histopathology and embedded in paraffin. Four µm-thick sections were examined after staining with haematoxylin and eosin (H&E) by a veterinary pathologist (S.To.) and by a board-certified veterinary pathologist (R.V.). A histopathological scoring system was developed based on currently available scientific literature [32, 34–37]. Briefly, the quality of the sections was assessed with a three-tier score, and samples were diagnosed as normal tissue, hyperplasia, gastrointestinal intraepithelial neoplasia (GIN)/adenoma/adenocarcinoma according to the guidelines published by [35]. Distribution was assessed for both hyperplastic and neoplastic lesions, whilst only for neoplasia a thorough characterization including macroscopic growth pattern, tumor histotype, grade of dysplasia, mitotic count (mean number of mitoses in 10 high microscopic power fields equal to 2.37 mm^2^) and ulceration was performed. Colonic inflammation was evaluated for all samples morphologically characterizing the cellular type, the severity and the extent of the inflammatory process as well as tissue edema. Finally, gastrointestinal-associated lymphoid tissue hyperplasia was assessed in all cases.

### 53BP1 immunohistochemistry

The samples were deparaffinized and antigen retrieval was achieved by soaking the slides in a pH 9 commercial solution (Envision FLEX High pH; Agilent Technologies Italia, ) for 60 min at 97 °C. The sections were then rinsed with PBS and a primary rabbit polyclonal antibody against 53BP1 was applied (see Supplementary Table 1) in a moist chamber at 4 °C with an overnight incubation period. The samples were then rinsed in PBS and a secondary HRP-conjugated goat anti-rabbit antibody (Abcam) was applied for 1 h at RT. After PBS rinsing DAB + liquid (Agilent Technologies Italia) was applied for 8 min at RT to assess 53BP1 colorimetric expression. Finally, the samples were counterstained with haematoxylin and dehydrated, and the cover slides were applied. The score for 53BP1 staining was graded as follows: The percentage of positively stained epithelial cells was scored in 4 grades: 1 (0 − 25%), 2 (25 − 50%), 3 (50 − 75%) and 4 (75 − 100%). The percentage of positively stained epithelial cells was scored after counting 5 microscopic fields at 20X.

### Multiplex immunofluorescence staining

Two multiplex fluorescence panels targeting myeloid and lymphoid cell lineages were selected and performed on 5 μm sections of FFPE mouse colon tissue. Detailed information on the antibodies, including clone, dilution and retrieval buffers, is provided in Supplementary Table 2. Multiplex IHC staining was performed using the Opal™ 7-Color IHC Kit (PerkinElmer) on the Leica BOND RX automated immunostainer (Leica MicrosystemsNuclei were counterstained with DAPI prepared according to the manufacturer’s instructions.

Fluorescence images were acquired using the Phenominager Automated Quantitative Pathology Imaging System (Akoya Biosciences) at 20× magnification.

Spectral unmixing, cell segmentation and phenotype classification based on marker expression were performed using inForm software v2.6.0 (Akoya Biosciences). A selection of representative multispectral images was used to train the inForm software to create algorithms. Cell phenotyping was based on the detection of co-localized cell surface. Cell density and percentage data were reported as the mean of all acquired fields from the same tissue slide at 20X magnification for each stained slide. Count and density analysis were calculated using phenoptrReports (add-ins for R Studio from Akoya Biosciences). QuPath version 0.5.1 software was used to create composite images of the entire tissue section from the images processed by inForm.

### Real time PCR

Colon tissue fragments were stored in RNAlater (Qiagen) at -20 °C. Total RNA was purified by TRI Reagent extraction (Zymo Research), followed by LiCl precipitation to remove DSS from colonic RNAs. cDNA templates were obtained by reverse transcription (Tetro cDNA Synthesis Kit), according to manufacturer’s instructions. Quantitative real-time PCR amplifications were performed in triplicate with SensiFAST™ SYBR^®^ Lo-ROX Kit (Meridian Bioscience), using Applied Biosystems 7500 Fast Real-Time PCR System. In order to verify the amplification of a single product, a melting curve was generated at the end of every run. The relative expression levels were calculated by the comparative cycle threshold (ΔΔC_T_) method and were normalized by the expression of ribosomal protein lateral stalk subunit P0 (Rplp0), a stably expressed housekeeping gene in our sample set. The primers for *Rplp0*, *Muc-2*, *Il1b*, *Il6*, *Il8*, *Il10*, *Ifng* and *Tnfa* were synthetized by Eurofins Genomics (Supplementary Table 3).

### Fecal DNA isolation, 16 S rRNA gene sequencing and taxonomic analysis

Pools of fresh fecal pellets collected from mice were used to isolate bacterial DNA, using the QIAamp Fast DNA Stool Mini Kit (Qiagen) according to the manufacturer’s instructions. DNA was checked for concentration and purity using a NanoDrop spectrophotometer and stored at -20 °C until further use. The fecal microbiota analysis was performed by 16 S rRNA gene sequencing, following the 16 S Metagenomics Sequencing Workflow provided by Illumina. Briefly, the V3-V4 region of the bacterial 16 S rRNA gene was amplified using the primers selected by Klindworth [38] modified with specific Illumina adapter sequences. Amplicons were purified, barcoded with dual-index system (Nextera XT Index Kit set A, Illumina) to allow a multiplex analysis, equimolarly pooled and subjected to paired-end sequencing (2 × 300 cycles) on a MiSeq platform (Illumina). De-multiplexed FASTQ files containing raw data were analysed using the 16 S Metagenomics GAIA 2.0 software (https://metagenomics.sequentiabiotech.com/gaia/: Sequentia Biotech). The software performs quality check (i.e., trimming, clipping and adapter removal) of the reads using FastQC and BBDuk, then it uses BWA to map quality-filtered sequences against the NCBI 16 S reference database for taxonomic assignments. Within-sample diversity, expressed by the Shannon evenness index and the Chao1 richness index, was also computed by the software. Pairwise differential abundance analyses were performed according to the DESeq2 statistics. Results were considered significant when *P* < 0.05 and FDR < 0.05.

### Statistical analysis

Unless otherwise specified, data were expressed as mean ± SD. Statistical significance was calculated by one-way ANOVA with post hoc testing (Tukey) and by Kruskal-Wallis ANOVA tests (GraphPad Prism 8). For the analysis of anaphases and metaphases, Fisher’s test was applied (R 4.3.3). For gene expression analysis, non-parametric Mann-Whitney U test was applied. *P* values less than 0.05 were considered as statistically significant.

## Results

### CNF1 toxin interferes with intestinal epithelial cell growth by inducing G2/M cell cycle arrest

**Fig. 1:**
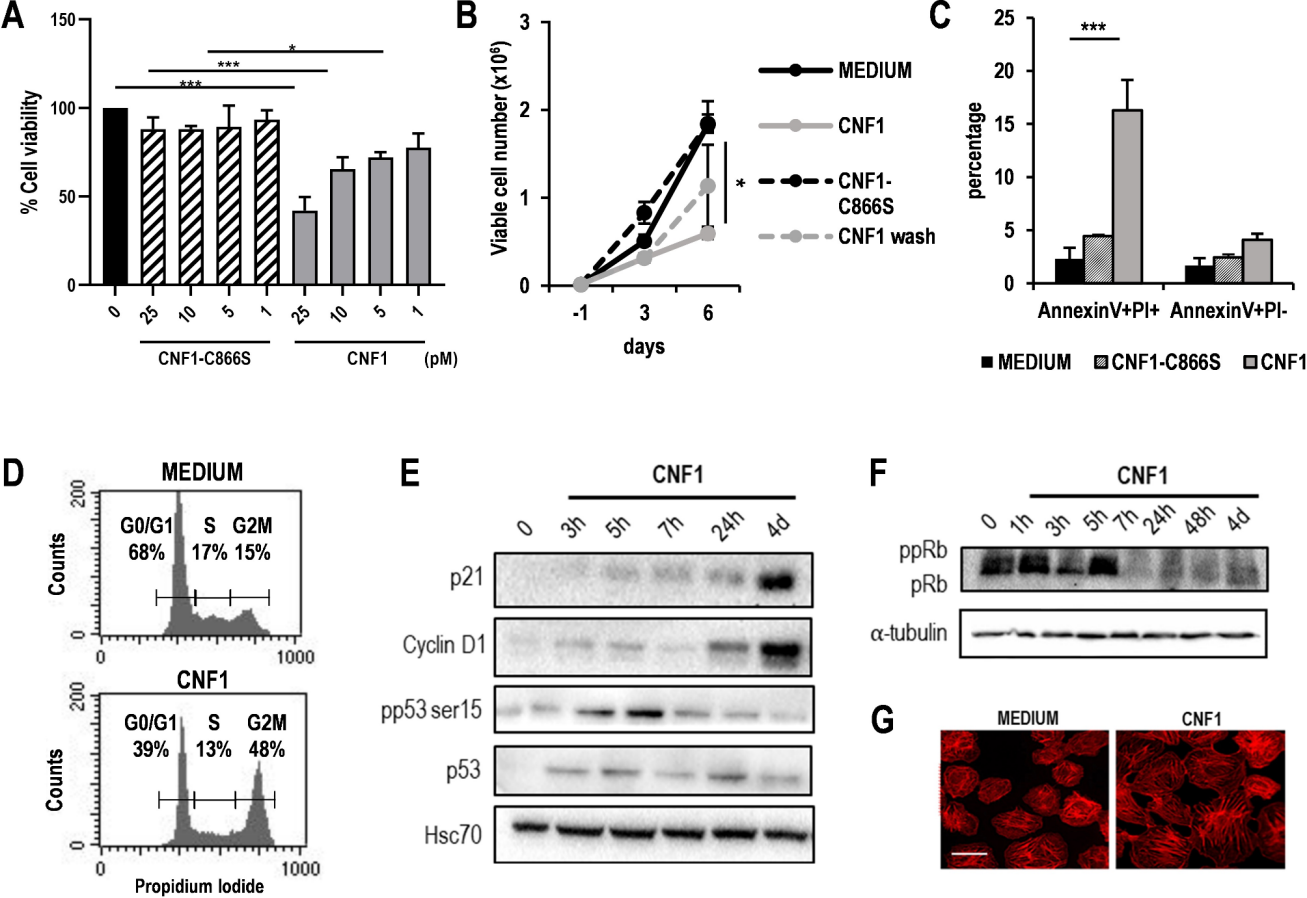
CNF1 affects epithelial cell growth. (**A**) MTT assay of IEC-6 cells after 6 days of culture in the presence of scalar concentrations of CNF1 or CNF1-C866S. One representative experiment out of two is shown. (*p < 0.05; ***p < 0.001). (**B**) Trypan blue exclusion count of IEC-6 after 3 and 6 days of culture in the presence of the indicated concentrations of CNF1 or CNF1-C866S (*N* = 3). One representative experiment out of three is shown. (*p < 0.05 vs. CNF1-C866S and PBS). (**C**) Percentage of Annexin V and/or PI-positive cells after 6 days of culture in the presence of CNF1 or CNF1-C866S (25 ρM) (*N* = 3). One representative experiment out of three is shown. (***p < 0.001 vs. CNF1-C866S and PBS). (**D**) Histogram plot of the cell cycle distribution of untreated IEC-6 cells or IEC-6 cells exposed to 25 ρM CNF1 for 24 h. One representative experiment out of three is shown. (**E**) Western blot analysis of p21, Cyclin D1, p53 and phosphorylated p53 (pp53), in cell lysates from untreated and CNF1-treated IEC-6 cells at different time-points from treatment. One representative experiment out of two is shown. (**F**) Western blot analysis of the hypophosphorylated (pRB) and hyperphosphorylated (ppRB) forms of RB in cell lysates from untreated and CNF1-treated IEC-6 cells for the indicated times. (**G**) Representative fluorescence micrographs of F-actin staining in untreated and CNF1-treated IEC-6 24 h after treatment. Scale bar, 10 μm

In a previous study, we demonstrated that CNF1 affects the proliferation of an uroepithelial cell line through the activation of Rho-GTPases [39]. In the present study, as a first step, we evaluated the effects of CNF1 on intestinal cells, which are the natural primary target of the toxin. To this aim, we cultured IEC-6 cells with decreasing concentrations of CNF1 (25 − 1 pM) for 6 days and evaluated cell viability by MTT assay. We observed a dose-dependent reduction of cell viability in CNF1-treated IEC-6 cells, as compared to untreated controls (Fig. 1A). This effect was dependent on Rho-GTPase activation as IEC-6 cells treated for 6 days with the same concentrations of CNF1-C866S, a variant of CNF1 toxin, which is unable to activate Rho-GTPases, did not modify their viability rates (Fig. 1A). Trypan blue exclusion count further confirmed these results. In fact, IEC-6 cells exposed to 25 pM CNF1 displayed a reduction of viable cell number, as compared to CNF1-C866S-treated cells or to their untreated controls (Fig. 1B). Interestingly, if CNF1 was removed from culture wells after 24 h and replaced with fresh medium, IEC-6 cells recovered their proliferative capacity, implying a reversible antiproliferative effect of the toxin (Fig. 1B). Annexin-V/PI staining of IEC-6 cells after 6 days of culture in the presence of CNF1 or CNF1-C866S revealed that less than 20% of cells had entered the apoptotic cell death program (Fig. 1C). HPCECs were also sensitive to the anti-proliferative effects of CNF1 as the number of viable cells after 6-days of culture in the presence of the toxin was significantly reduced, as compared to the same dose of CNF1-C866S or to the untreated controls (Supplementary Fig. 1A).

The observed growth arrest and the lack of massive cell death in IEC-6 cultures treated with CNF1 prompted us to investigate whether this toxin could interfere with regular cell cycle progression. Thus, IEC-6 cells were exposed for 24 h to CNF1 and the distribution through the different phases of the cell cycle was evaluated by flow cytometry. While in the control culture the majority of the cells (68%) were in G0/G1 phase, treatment with CNF1 toxin induced the accumulation of cells in G2/M phase (Fig. 1D), indicating cell cycle arrest. Consistently, WB analysis showed that CNF1-treated IEC-6 cells progressively upregulate the cyclin-dependent kinase inhibitor p21 and its transcriptional activator p53 (Fig. 1E). Toxin-treated cells also upregulate Cyclin D1 (Fig. 1E), a crucial regulator of cell cycle progression whose functional activation is tightly linked to p21 [40]. In addition, CNF1-exposed cultures accumulate the hypophosphorylated form of retinoblastoma protein (pRB) (Fig. 1F), a surrogate marker of the proliferative status of cells [41], which drives cell cycle exit by sequestering E2F family transcription factors.

Morphological analysis of both IEC-6 and HPCEC cells revealed that, coherently with previous data [28], CNF1 induced, in both cell types, large, flattened, and multinucleated cells (data not shown). At the same time, the actin cytoskeleton was remarkably reorganized with stress fibres, membrane ruffles and filopodia, as expected by the CNF1-induced activation of Rho-GTPases (Fig. 1G, Supplementary Fig. 1B). Altogether, these results indicate that CNF1 interferes with normal epithelial cell growth and morphology, and stimulates signalling pathways ultimately leading to G2/M cell cycle arrest.

### CNF1 induces genetic instability in intestinal epithelial cells in vitro

**Fig. 2:**
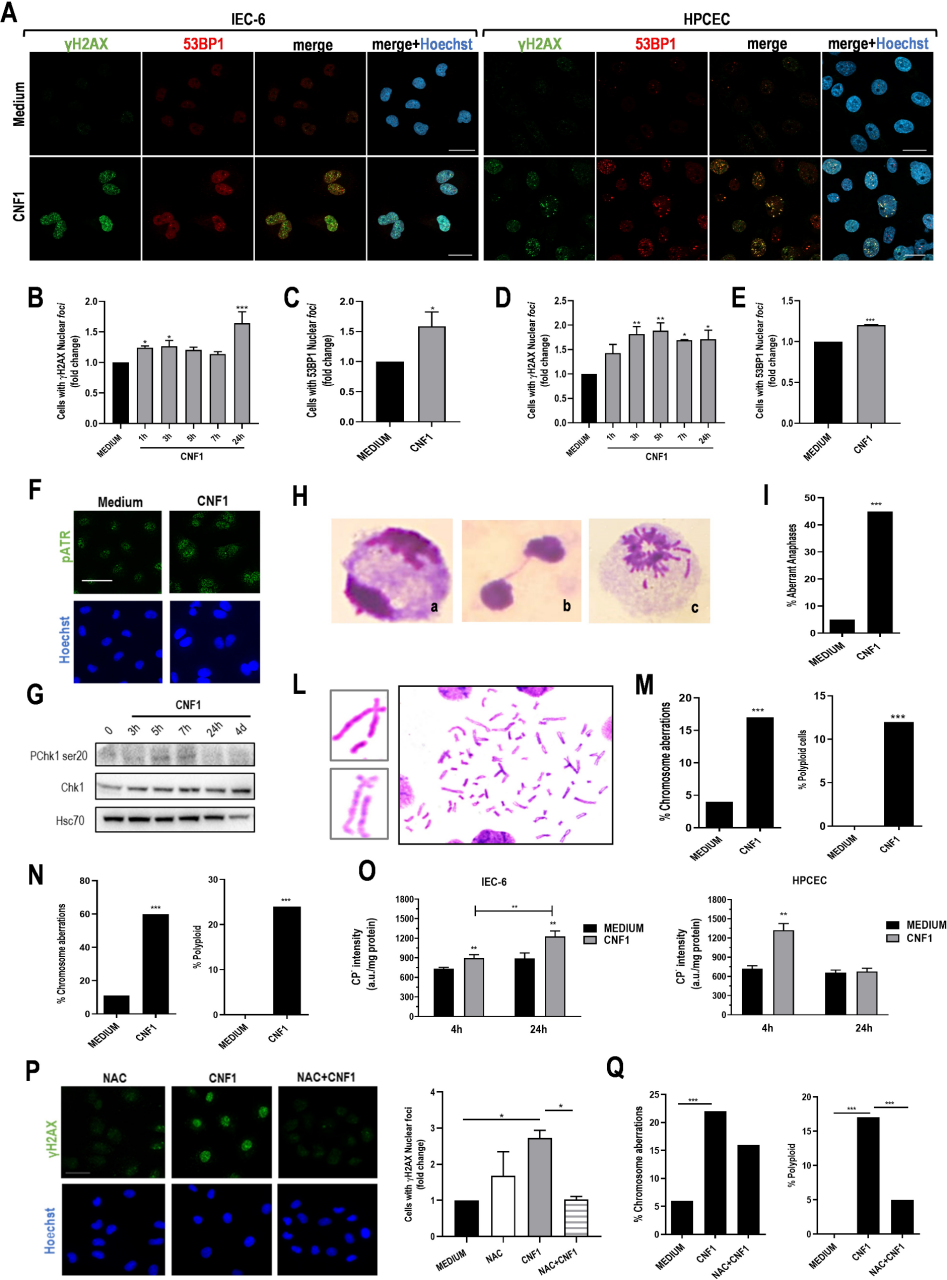
CNF1 induces oxidative stress and genetic instability in intestinal epithelial cells. (**A**) Representative CLSM micrographs of IEC-6 and HPCEC cells stained with anti-γH2AX (green) and anti-53BP1 (red) antibodies. Nuclei were counterstained with Hoechst (blue). Scale bars, 10 μm. (**B**) Bar plot showing fold change of γH2AX-positive nuclei and (**C**) bar plot showing fold change of 53BP1-positive nuclei in IEC-6 cells at different time points following exposure to CNF1. (**D**) Bar plot showing fold change of γH2AX-positive nuclei and (**E**) bar plot showing 53BP1-positive HPCEC nuclei at different time points following exposure to CNF1. One hundred nuclei at each experimental point were counted. (*p < 0.05; **p < 0.01; ***p < 0.001). (**F**) Representative fluorescence micrographs of IEC-6 cells stained with anti-pATR (green). Nuclei were counterstained with Hoechst (blue). Scale bars, 10 μm. (**G**) Western blot analysis of Chk1 and its phosphorylated form at the indicated time points. (**H**) Representative micrographs of Giemsa-stained IEC-6 nuclei showing: (**a**) normal anaphase; (**b**) bridge and (**c**) circular nucleus. (**I**) Bar plot showing the percentage of aberrant anaphases in untreated and in CNF1-treated IEC-6. One hundred nuclei for each experimental condition were analysed (***p < 0.001). (**L**) Representative micrographs and (**M**) bar plot showing the percentage of chromosome aberrations and polyploid cells in nuclei metaphases of IEC-6 cells treated with CNF1 as compared to untreated cells. (**N**) Bar plot showing the percentage of chromosome aberrations and polyploid cells after further 48 h of culture in the absence of CNF1 toxin. Two hundred metaphases were counted for each experimental condition (***p < 0.001). (**O**) Bar plots showing ROS concentration measured by EPR spectroscopy in IEC-6 and HPCEC cells exposed to 25 ρM CNF1 or to medium as control. (**P**) Representative fluorescence micrographs and bar plot showing the relative γH2AX amount in IEC-6 cells pre-treated with the anti-oxidant NAC (10 M) before CNF1 exposure. Nuclei are stained with Hoechst (blue). Scale bar: 10 μm. (*p < 0.05). (**Q**) Percentage of chromosome aberrations and polyploid cells of IEC-6 cells pre-treated with the anti-oxidant NAC before CNF1 exposure. Two hundred metaphases were counted for each experimental condition (***p < 0.001)

Since CNF1 activates p53 and blocks the cell cycle in G2/M (Fig. 1), which are typical features indicative of DNA damage, we investigated whether exposure of epithelial intestinal cells to CNF1 could induce DNA damage by analysing two markers of the DNA-damage response (DDR) pathway: the phosphorylated form of the H2AX (γH2AX), and the suppressor protein 53BP1. Fluorescence microscopy analysis (Fig. 2A-B-D) of IEC-6 and HPCEC cells cultured for different times in the presence of CNF1 showed a significant increase in the frequency of nuclei positive for γH2AX foci starting already 1–3 h after treatment in both cell types, as compared to control cultures. A more detailed analysis of the distribution of γH2AX foci highlighted a preferential increase of highly damaged cells (10 < γH2AX < 20 and > 20 foci/nucleus) 24 h after toxin exposure in IEC-6 cells and in HPCEC, although in the latter the results did not reach statistical significance (Supplementary Fig. 2A). The upregulation of γH2AX was also confirmed by WB analysis (Supplementary Fig. 2B-C). Consistently with the activation of H2AX, we also observed an increase in the percentage of nuclei positive for 53BP1 foci in IEC-6 and HPCEC cells after 24 h of treatment with CNF1, (Fig. 2A-C-E). Of note, no changes in the amount of γH2AX-positive nuclei were observed in IEC-6 cells exposed to the inactive form of the toxin, i.e., the mutant CNF1-C866S (Supplementary Fig. 2D), indicating that the induction of the DDR is dependent on Rho-GTPases activation. We additionally demonstrated the involvement of DDR through fluorescence and WB analyses, which showed that CNF1 increases the number of pATR foci (Fig. 2F) and phosphorylates CHK1 for at least up to 7 h of treatment (Fig. 2G).

The activation of DDR, demonstrated by the increase of γH2AX and 53BP1, prompted us to investigate further the nature of the CNF1-induced DNA damage by cytogenetic analysis of chromosomal damage in IEC-6 cells. At first, we analysed chromosome segregation by morphological inspection of anaphases after 24 h of treatment with CNF1 as compared to untreated cells (Fig. 2H-I). Interestingly, CNF1 induced a statistically significant increase of aberrant anaphases showing chromosome bridges between the two daughter nuclei (Fig. 2H, panel b), displaced chromosomes and donut-shaped nuclei (Fig. 2H, panel c). This evidence indicates that the toxin can indeed interfere with chromosome segregation in daughter cells. To investigate the type of chromosomal damage induced by CNF1, structural and numerical (i.e., polyploidy), chromosome aberrations were analysed in metaphase IEC-6 cells treated with CNF1 for 24 h. A statistically significant increase in the frequency of structural chromosome aberrations and polyploidy cells was observed in CNF1-treated cultures, as compared to the untreated cells (Fig. 2L-M). To evaluate the persistence of DNA damage, IEC-6 cells were treated with CNF1 for 24 h, then the toxin was removed and the cells were cultured in fresh medium for additional 48 h. Surprisingly, a statistically significant increase in chromatid breaks and in polyploid cells was still observed in CNF1-treated cells, suggesting that the damaged cells were not fully repaired nor had undergone a cell death program (Fig. 2N). Overall, these results indicate that CNF1 exerts a genotoxic activity on intestinal epithelial cells and that this can lead to genomic instability.

Since oxidative stress is known to be associated with the induction of oxidative DNA damage [41], and previous studies reported the effect of CNF1 on the structure and activity of mitochondria [25, 28] we evaluated whether the toxin could increase oxidative stress through the production of ROS. Thus, IEC-6 and HPCEC cells exposed to CNF1 were analysed by EPR-spectroscopy and, as reported in Fig. 2O, a statistically significant increase in the level of ROS was found 4 h after treatment in both cell types. The increase was maintained up to 24 h in the IEC-6 cell line.

To investigate the possible dependency of CNF1-induced genotoxicity (i.e., DDR activation, structural chromosomal damage, numerical chromosome damage/polyploidy) from ROS, we pre-treated IEC-6 cells with the antioxidant NAC for 2 h before CNF1 addition and evaluated CNF1-induced H2AX phosphorylation and chromosome aberrations. Fluorescence microscopy analysis revealed that NAC pre-treatment was able to prevent the formation of γH2AX foci in CNF1-treated cells keeping the number of foci at the level of the control (Fig. 2A and P), thus indicating that ROS are the main players in this phenomenon. On the contrary, NAC pre-treatment did not significantly reduce the frequency of chromosome aberrations in metaphases of CNF1-treated IEC-6 cells, while it only decreased the frequency of polyploid cells (Fig. 2Q).

Altogether, these data indicate that CNF1 induces oxidative stress, chromosome aberrations and polyploidy. However, while CNF1-induced polyploidy is dependent on ROS, the structural chromosome aberrations observed are independent.

### CNF1 induces intestinal barrier permeabilization

**Fig. 3:**
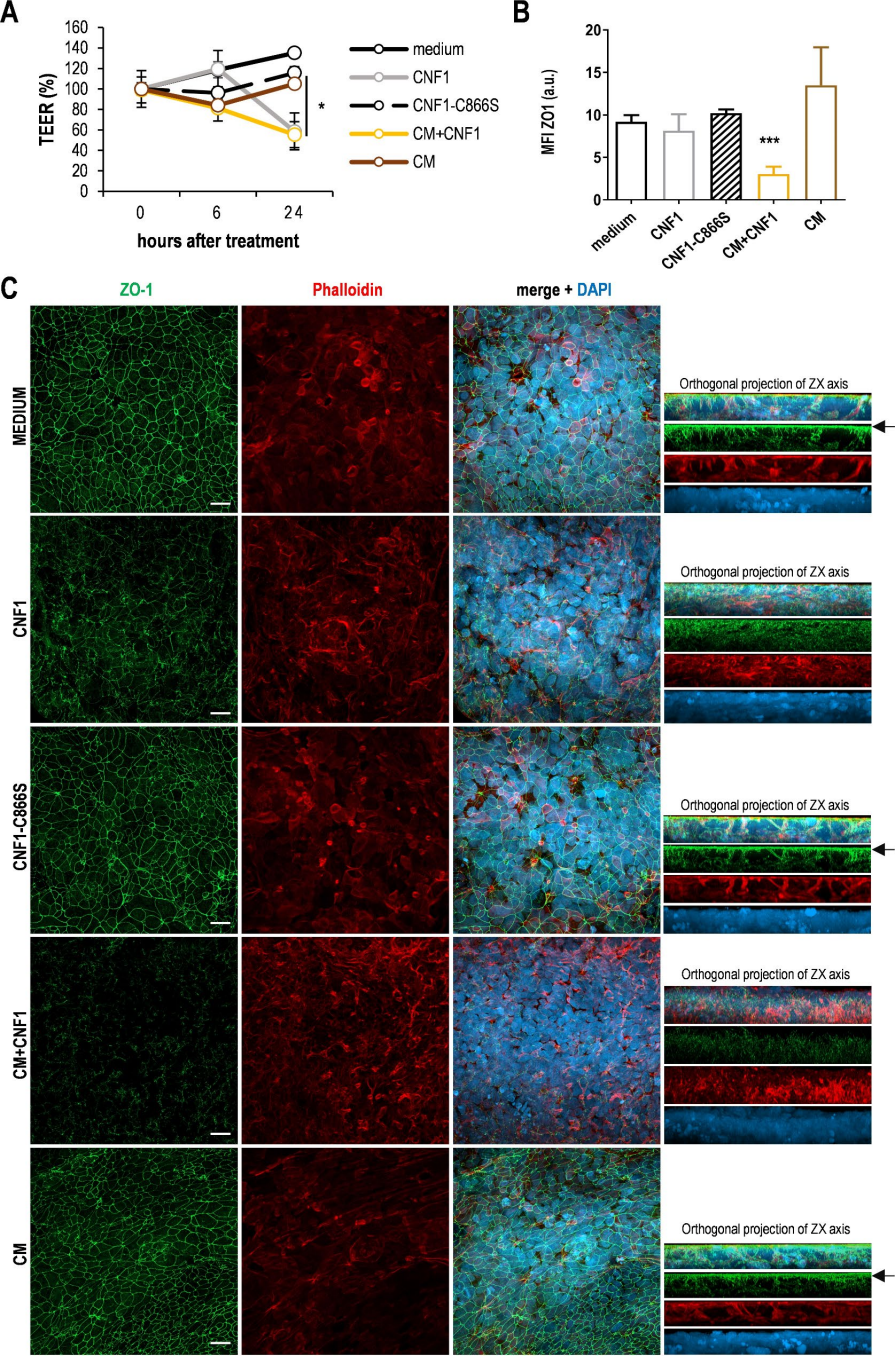
CNF1 impairs gut barrier integrity. (**A**) Changes of Trans-Epithelial Electrical Resistance (TEER) in Caco-2 monolayers cultured for 21 days on 3 μm pore size inserts and exposed for the indicated times to CNF1 or CNF1-C866S (25 pM) or a conditioned medium (CM) from activated THP-1 cells or CM + CNF1. Data are expressed as percent TEER changes compared to the TEER value registered before treatment (day 0). *N* = 4. (*p < 0.05). (**B**) Mean fluorescence intensity (MFI) of ZO-1 expression in differentiated Caco-2 monolayers 24 h after the indicated treatments. *N* = 4. (***p < 0.001). (**C**) Representative micrographs from CLSM examinations (3D reconstruction images). Cell monolayers were stained with anti ZO-1 (green) and phalloidin (red) to visualize actin proteins. Nuclei are stained with DAPI (blue). Separate channels and merged images are shown. On the right, orthogonal projections of transverse ZX axis of the same image are reported. Scale bars, 20 μm. Images from one representative experiment out of three are shown

**Fig. 4:**
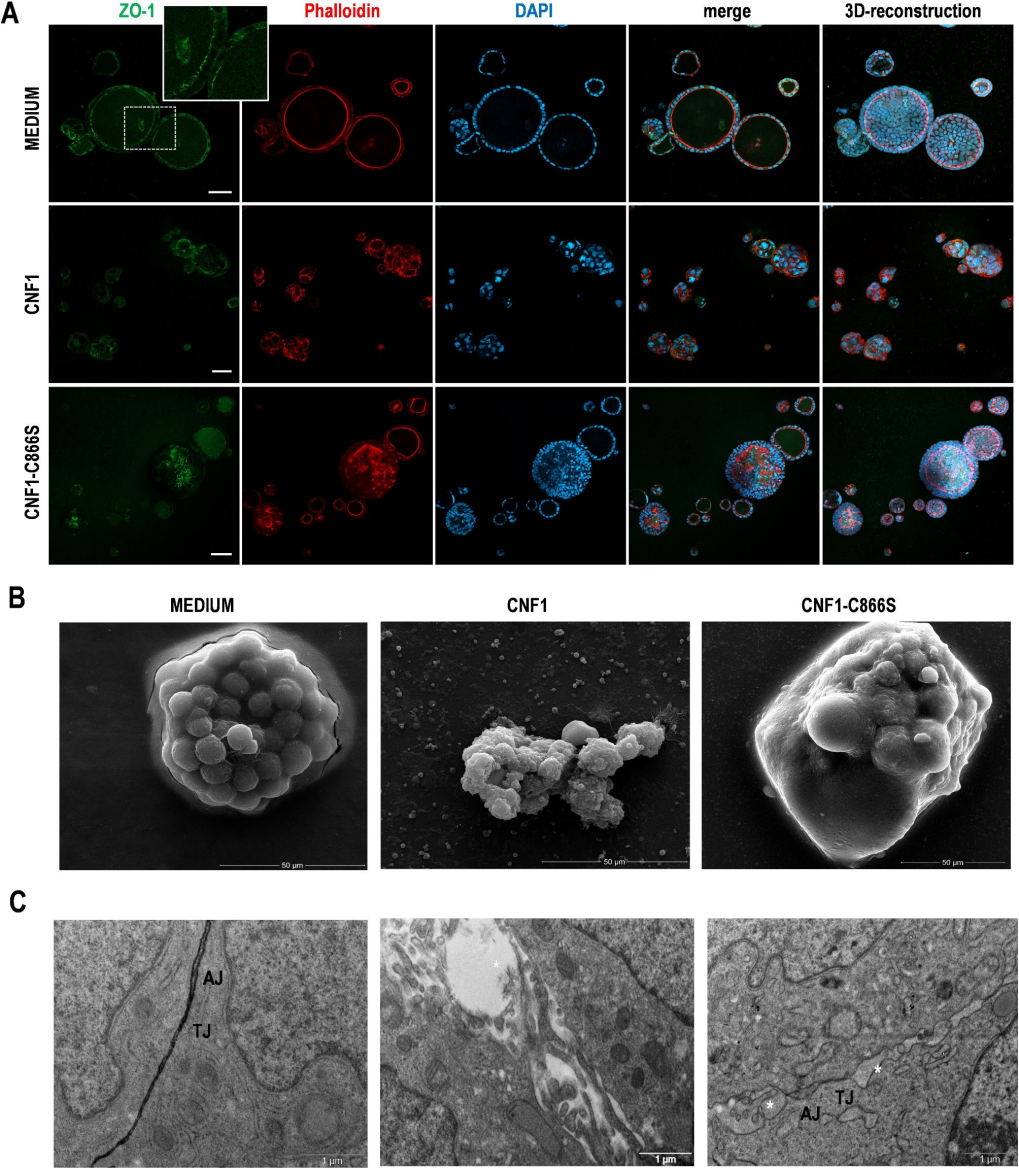
CNF1 alters 3D Caco-2 spheroids formation and disrupts cellular junctions. (**A**) Representative images from CLSM examinations (central optical sections and 3D reconstruction images). 3D Caco-2 spheroids were stained with anti-ZO-1 (green) and phalloidin (red) to visualize actin proteins. Nuclei are stained with DAPI (blue). Separate channels and merged images are shown. In the insert of control spheroids a higher magnification of ZO-1 distribution is shown. 3D reconstructions of the entire spheroids are reported on the right. Scale bars, 50 μm. Representative examples of 3 independent experiments are shown. (**B**) Representative scanning electron microscopy micrographs of Caco-2 spheroids grown in control medium, or in presence of CNF1 and CNF1-C866S toxins, showing their 3D overall spatial architecture comprehensive of the surface features. (**C**) Representative transmission electron microscopy micrographs of Caco-2 spheroids cultured in control medium, or in presence of CNF1 and CNF1-C866S toxins showing their internal ultrastrucural organization (TJ = tight junction; AJ = adherens junction; * Intercellular spaces)

Numerous investigations report the association between gut permeability alterations and intestinal or extra-intestinal disorders, including cancer. To evaluate whether CNF1 can induce epithelial leakiness, we established a long-term culture of Caco-2 cells, a well-characterized cellular model that recapitulates gut morphology and permeability characteristics. Exposure of Caco-2 monolayers to CNF1 induced a time-dependent halving of TEER, as compared to control monolayers (Fig. 3A). This effect was paralleled by an altered distribution of ZO-1 protein and loss of the typical architecture of tight junctions (TJ), as observed by confocal microscopy (Fig. 3B-C). Interestingly, CNF1-induced permeabilization of Caco-2 monolayer was strongly enhanced by the addition of pro-inflammatory factors (Conditioned Medium, CM), which also induced a significant down-regulation of ZO-1 expression, with the complete disruption of TJ architecture (Fig. 3A-C). The activity of CNF1 was also investigated by using an advanced in vitro 3D intestinal model, i.e., 3D Caco-2 spheroids, able to replicate, in a more realistic manner, traits of in vivo intestinal epithelium including a single central hollow lumen [42]. As shown by confocal microscopy investigations, Caco-2 cells grown in control medium formed hollow, fully-differentiated 3D spheroids characterized by a single layer of cells surrounding a central hollow lumen with the typical spatial arrangement of nuclei, and F-actin ring and ZO-1 regularly outlining their inner lumen (Fig. 4A). In contrast, Caco-2 cells cultured in the presence of CNF1 formed cell aggregates in which F-actin and ZO-1 were randomly distributed. In Caco-2 spheroid cultures obtained in the presence of CNF1-C866S toxin, well-formed 3D spheroids were detectable together with undifferentiated spheroids lacking a central hollow lumen in which ZO-1 and F-actin are randomly expressed. Accordingly, SEM studies (Fig. 4B) showed that control Caco-2 spheroids indeed appeared completely formed, well rounded and with single cells uniform in shape, whereas CNF1 treatment induced the formation of 3D structures composed of cells with evident signs of damage. In CNF1-C866S-treated cultures, 3D Caco-2 spheroids characterized by non-uniform shape were observed. These results were further strengthened by TEM analysis (Fig. 4C) showing that, in control spheroids, cells appear intimately associated with each other and closely packed, and tight junctions and adherent junctions can be plainly identified between cells. In contrast, in CNF1-treated cultures aggregates formed by loosely associated cells with apparent intercellular spaces are evident. In CNF1-C866S-treated cultures, areas with highly associated cells together with zones where cells are much less packed, and an inequality of junctional complex distribution are clearly detectable. Altogether, these data indicate that CNF1 directly alters permeabilization in in vitro 2D model and hinders the proper formation of differentiated Caco-2 spheroids, involving the irregular distribution of TJ proteins and, thus, influencing the overall permeabilization status.

### CNF1 induces colorectal tumors in vivo

**Fig. 5:**
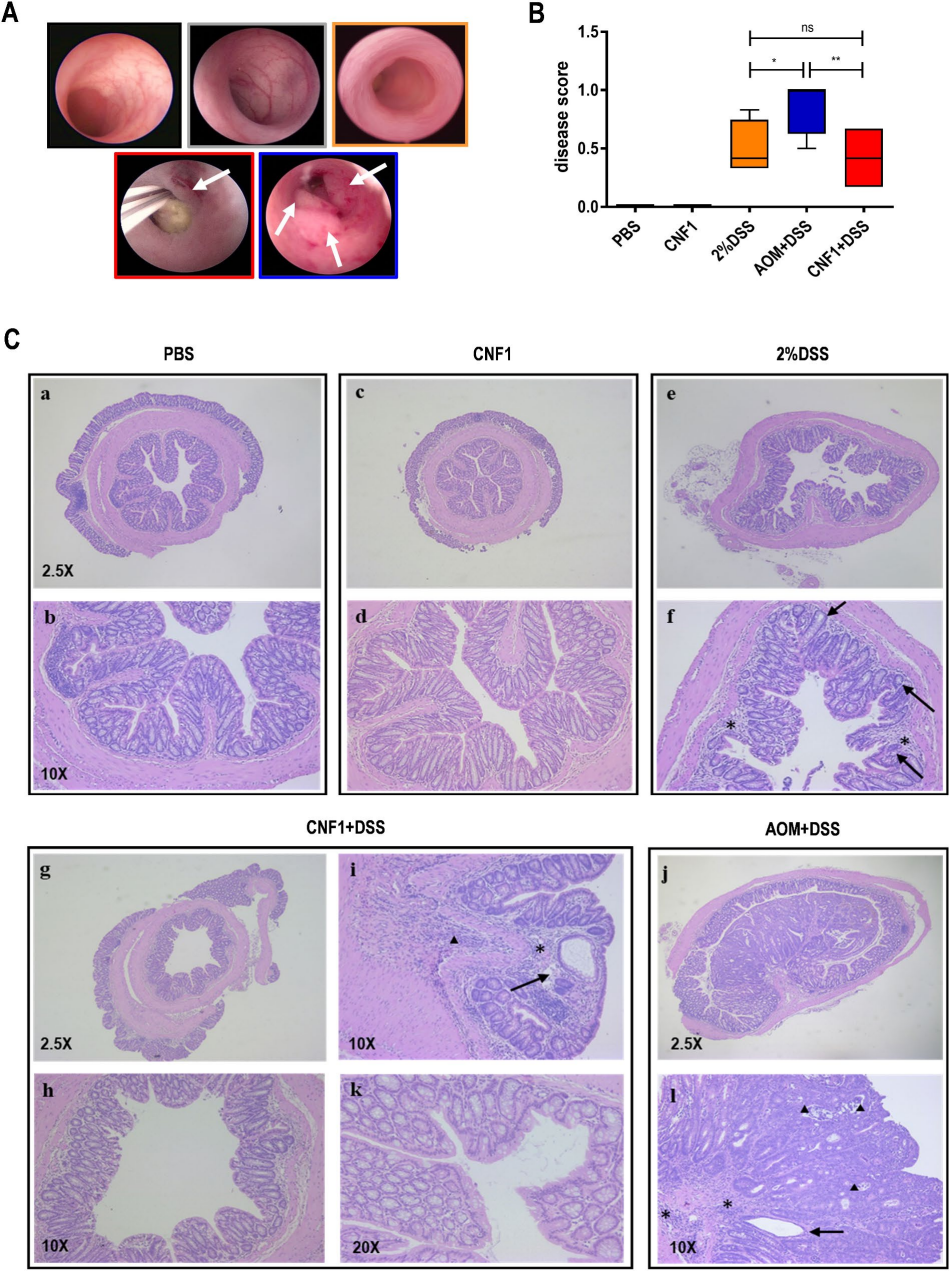
Morphological analysis of colon tissue after three cycles of treatment. (**A**) Representative high-resolution colonoscopy images from the indicated treatment groups. Arrows indicate tumor lesions and mucosal thickening. (**B**) Boxplot depicting the disease score generated at colonoscopy. Center lines show the medians; box limits indicate the 25th and 75th percentiles; whiskers represent Min and Max values. (ns = non significant; *p < 0.05; **p < 0.01; ***p < 0.001). (**C**) H&E staining of FFPE colon sections: (**a-c**) Normal colonic tissue; (**b-d**) Higher magnification of pictures a-c; (**e**) Hyperplastic colonic mucosa thrown up in multifocal folds; (**f**) Higher magnification of picture (**e**) with evidence of mucosal thickening (arrows) and inflammatory infiltrates in the lamina propria (asterisk); (**g**) Hyperplastic mucosa and (**h**) higher magnification of the same sample; (**i**, **k**) focal intestinal crypt ectasia and moderate mixed inflammation within the lamina propria (asterisk) and the submucosa (arrowhead); (**j**) focal adenoma growing within and partially obliterating the intestinal lumen. (**l**) higher magnification of picture (**j**) showing focal new formed crypt ectasia (arrow), inflammation in the submucosa and lamina propria (asterisk) and multifocal crypt abscesses (arrowhead)

Since previous data evidenced a cooperation between inflammatory signals and CNF1 in the intestinal barrier permeabilization (Fig. 3A-C) and in the upregulation of epithelial mesenchymal transition markers [43], we adapted a well-characterized inflammatory carcinogenesis model (AOM/DSS model) to verify the existence of a causal link between CNF1 and CRC. To this aim, AOM injections in the AOM/DSS model were replaced by monthly i.r. administrations of CNF1 followed by DSS cycles (2% in the drinking water), as summarized in Supplementary Fig. 3A. As expected, all the groups that received DSS experienced a transient weight loss, which was more pronounced after the first cycle of treatment, while the mice treated with CNF1 alone did not experience any weight loss (Supplementary Fig. 3B). On day 70 and 154 after treatment, all animals underwent colonoscopy to identify any morphological changes induced by CNF1 in vivo. Morphological characteristics of the colonic mucosa concurred to generate a disease score that was calculated according to the parameters and scores reported in Supplementary Table 4. After three cycles of treatment (day 70), PBS and CNF1-treated animals had a smooth and transparent mucosa with the normal vascular pattern, as testified by a disease score equal to 0 (Fig. 5A-B). Instead, mice treated with 2% DSS and with CNF1 + DSS had a worsening of the disease score, confirming the development of an IBD (Fig. 5A-B). Histological examination of colonic tissue from these treatment groups revealed a mucosal thickening and the presence of inflammatory infiltrates (Fig. 5C). Of note, in mice treated with CNF1 + DSS, few intestinal crypt ectasias were also documented (Fig. 5C, panel g-k). As expected, mice treated with AOM/DSS showed a further worsening of the disease score and several tumor lesions clearly visible by colonoscopy and histologically confirmed (Fig. 5B-C).

**Fig. 6:**
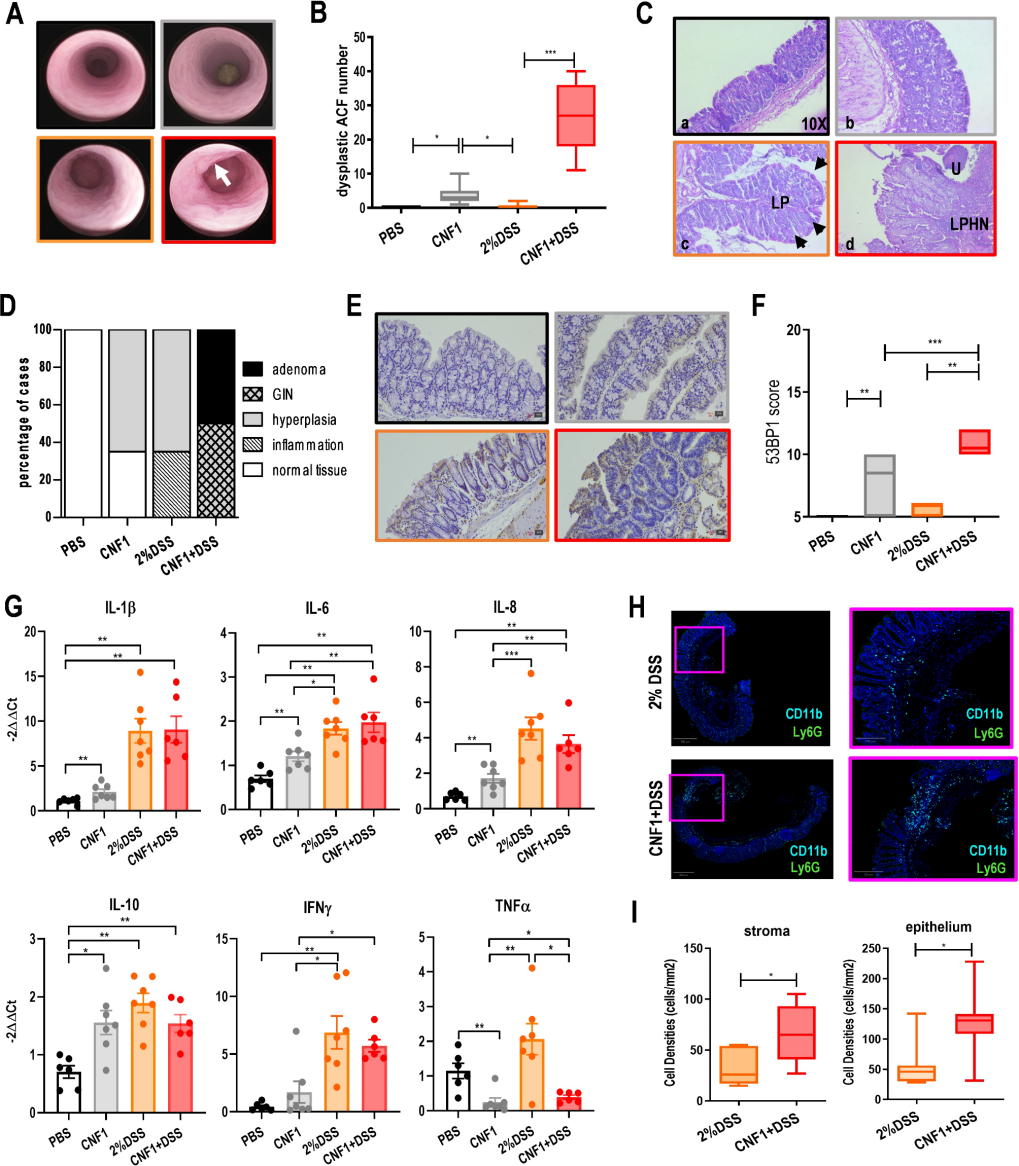
Colorectal tumorigenesis in DSS–treated mice after six cycles of i.r. CNF1 administration. (**A**) Representative high-resolution colonoscopy images from the indicated treatment groups (*black square*, PBS; *grey square*, CNF1; *orange square*, DSS; *red square*, CNF1+DSS). Arrow indicates a macroscopic tumor lesion. (**B**) Boxplot depicting the number of dysplastic aberrant crypt foci (ACF) in colon sections from the indicated treatment groups (*N* = 12 per group). Center lines show the medians; box limits indicate the 25th and 75th percentiles; whiskers represent Min and Max values. (*p < 0.05; ***p < 0.001). (**C**) Representative micrographs of H&E stained colonic sections: (*a*) Normal colon; (*b*) diffuse mild-to-moderate hyperplasia of mucosa; (*c*) diffuse moderate hyperplasia of the mucosa that is thrown up in folds (arrows). The lamina propria shows mild lymphoplasmacytic inflammation (LP); (*d*) adenoma with low grade atypia and a presence of ulceration (U) and diffuse mixed lymphoplasmacytic, histiocytic, neutrophilic (LPHN) inflammation. (**D**) Barplot indicating the distribution of the histopathological features of the colon tissue among the different treatment groups (*N* = 8 per group). (**E**) Representative micrographs of 53BP1 nuclear expression in colonic sections from the indicated treatment groups. (**F**) Boxplot depicting 53BP1 staining score. Center lines show the medians; box limits indicate the 25th and 75th percentiles; whiskers represent Min and Max values. (**p < 0.01; ***p < 0.001). (**G**) Barplots indicating gene expression by real-time PCR of the indicated cytokines in colon tissue. Data are expressed as mean ± SEM. (*p < 0.05; ** p < 0.01; ***p <0.001)*.*   (**H**) Representative micrographs of double stained CD11b+ (light blue) and Ly6G+ (green) cells (i.e. neutrophils) in colonic sections from the indicated treatment groups analyzed by multiplex IF imaging. (**I**) Boxplot showing the density of neutrophils (CD11b + Ly6G+) in stroma and epithelium compartments of colon tissue from the indicated treatment groups (*N* = 7 per group). Center lines show the medians; box limits indicate the 25th and 75th percentiles; whiskers represent Min and Max values. (*p <0.05)

After six months of treatment (day 154), mice in the CNF1 + DSS group showed transparent and fragile mucosa with macroscopically altered vascular pattern and, in some cases, protruding lesions (Fig. 6A). All these alterations reflected in the higher disease score of CNF1 + DSS-treated mice as compared to the single treatments (Supplementary Fig. 3C). It is interesting to note that mice exposed to CNF1 alone had a statistically significant increase of the disease score with respect to control animals, suggesting a mild perturbation of the gut equilibrium induced by the toxin (Supplementary Fig. 3C). This was paralleled in both CNF1- and CNF1 + DSS-treated mice by a significant increase of *Muc2* gene expression (Supplementary Fig. 3D), encoding for the major constituent of the colon mucus that protects the gut lumen against particles and infectious agents. Since intestinal permeability alterations are associated with IBD and may play a role in CRC onset, we performed an in vivo permeability assay to evaluate the effects of each treatment on intestinal epithelium integrity. As expected, in DSS-treated animals, colonic permeability to dextran-FITC was significantly increased with respect to PBS or to CNF1-treated animals (Supplementary Fig. 3E), confirming that DSS induces an intestinal barrier dysfunction [44]. Addition of CNF1 to DSS slightly increased intestinal permeability with respect to DSS alone, although the results did not reach statistical significance (Supplementary Fig. 3E). Histological examination of colonic tissues from all groups revealed the presence of a higher number of dysplastic ACF, i.e., precursors of grossly visible neoplastic lesions, in colonic sections from CNF1 + DSS-treated animals, as compared to DSS only group (Fig. 6B). Most importantly, while only diffuse inflammation and hyperplastic changes were reported in animals treated with either DSS or CNF1 alone (Fig. 6C-D), combined CNF1 + DSS-treatment caused the formation of GIN, squamous metaplasia and, in 50% of cases, colon adenomas (Fig. 6C-D), whose pathological features are reported in Table 1. Namely, adenomas were characterized by focal proliferation of mucosal epithelial cells with a broad-based growth organized in newly formed tubular structures protruding towards the intestinal lumen. Neoplastic cells showed low grade atypical features and a low mitotic count and no evidence of local invasion. Of note, staining of colon sections with anti-53BP1 showed an increase in the nuclear expression of this marker in CNF1 + DSS-treated animals as compared to DSS-treated and an increase in CNF1-treated animals, as compared to PBS, indicating a higher rate of double-strand break (DSB) in CNF1-treated colonic mucosa as compared to controls (Fig. 6E-F). Since both DSB and DSS-induced tissue damage can trigger the release of pro-inflammatory cytokines, we analyzed by real-time PCR the cytokine milieu induced by single and combined treatments. Interestingly, the gene expression of both pro-inflammatory (such as *Il1b*, *Il6* and *Il8*) and anti-inflammatory (such as *Il10*) mediators was significantly increased by CNF1 and DSS alone as well as by CNF1 + DSS treatment with respect to PBS (Fig. 6G), indicating that at this time point (day 154) (Supplementary Fig. 3A) both activation and resolution of the inflammatory response to injury are taking place. In addition, *Ifng*, which plays a critical role in initiating and maintaining inflammation in DSS-induced colitis, was significantly up-regulated in DSS and CNF1 + DSS-treated animals but was not induced by CNF1 (Fig. 6G). A different pattern of gene expression was observed for *Tnfa* that was decreased by CNF1 treatment as compared to PBS as well as by the combined treatment with respect to DSS alone (Fig. 6G), most likely indicating that the resolution of the inflammatory response is faster upon CNF1 than DSS treatment.

**Table 1:** Histopathological analysis of colon samples

Sample	Diagnosis	Hyperplasia/GIN	Adenoma/Adenocarcinoma						Inflammation				GALT
		Distribution	Macroscopic growth pattern	Herniation (Y/*N*)	Histotype	Grade of dysplasia (high/low)	Mitotic count	Ulcer (Y/*N*)	Type	Severity (1–3)	Extent	Edema (0–3)	Hyperplasia (0–3)
**PBS_1**	Normal								L	1	Diffuse	0	0
**PBS_2**	Normal								L	1	Diffuse	0	0
**PBS_3**	Normal								L	1	Diffuse	0	0
**PBS_4**	Normal								L	1	Diffuse	0	0
**PBS_5**	Normal								L	1	Diffuse	1	0
**DSS_1**	Inflamation	Diffuse							LP	1	Diffuse	1	2
**DSS_2**	Inflamation/Hyperplasia	Diffuse							LP	1	Diffuse	0	2
**DSS_3**	Inflamation/Hyperplasia	Diffuse							LP	2	Diffuse	1	2
**DSS_4**	Inflamation	Diffuse							LP	2	Diffuse	1	2
**DSS_5**	Inflamation/Hyperplasia	Diffuse							LP	2	Diffuse	1	2
**DSS_6**	Inflamation/Hyperplasia	Diffuse							LP	1	Diffuse	1	1
**DSS_7**	Inflamation/Hyperplasia	Diffuse							LP	1	Diffuse	1	1
**DSS_8**	Inflamation	Diffuse							LP	1	Diffuse	2	1
**CNF1/DSS_1**	GIN + Sq. metaplasia	Diffuse (GIN) focal (Metaplasia)		N					LPH	2	Diffuse	2	2
**CNF1/DSS_2**	Adenoma + GIN	Diffuse (GIN) focal (adenoma)	broad-based	N	Tubular	low	15	Y	LPHN	2	Diffuse	2	1
**CNF1/DSS_3**	Adenoma + GIN	Diffuse (GIN) focal (adenoma)	broad-based	N	Tubular	low	9	N	LPHN	2	Diffuse	1	1
**CNF1/DSS_4**	GIN	Diffuse		N					LPH	2	Diffuse	1	2
**CNF1/DSS_5**	GIN	Diffuse		N					LPH	2	Diffuse	1	2
**CNF1/DSS_6**	GIN + Adenoma	Diffuse + Focal (adenoma)	broad-based	N	Tubular	low	5	N	LPH	3	Diffuse	1	2
**CNF1/DSS_7**	Adenoma	Focal	broad-based	N	Tubular	low	5	N	LPH	2	Diffuse	2	2
**CNF1/DSS_8**	GIN + Sq. metaplasia	Diffuse		N				Y	LPHN	2	Diffuse	2	2
**CNF1_1**	Normal								L	1	Diffuse	1	0
**CNF1_2**	Hyperplasia	Diffuse							LP	1	Diffuse	1	0
**CNF1_3**	Hyperplasia	Diffuse							LP	1	Diffuse	1	0
**CNF1_4**	Hyperplasia	Diffuse							LP	1	Diffuse	1	0
**CNF1_5**	Hyperplasia	Diffuse							LP	1	Diffuse	1	0
**CNF1_6**	Normal								L	1	Diffuse	1	0
**CNF1_7**	Normal								L	1	Diffuse	1	0
**CNF1_8**	Hyperplasia	Diffuse							LP	1	Multifocal	1	1

Legend: GIN = gastrointestinal intraepithelial neoplasia; inflammation type L = lymphocytic, LP = lymphoplasmacytic, H = histiocytic, N = neutrophilic; inflammation severity 1 = mild (scarse inflammatory cells confined to the lamina propria), 2 = moderate (moderate inflammatory cells confined to the lamina propria), 3 = severe (inflammatory cells involving the submucosa); edema 0 = absent, 1 = mild (confined to the lamina propria), 2 = moderate (confined to the lamina propria), 3 = severe (involving the submucosa); gastrointestinal-associated lymphoid tissue (GALT) hyperplasia 0 = absent, 1 = mild, 2 = moderate, 3 = severe

Since inflammatory and immune-suppressive immune infiltrates are a well-established feature of colitis-associated CRC, we further characterized the type of immune infiltrate in colon tissue from CNF1 + DSS-treated with respect to DSS-treated animals, by multiplex spatial immunofluorescence staining of FFPE sections. To this aim, we designed two antibody panels to identify the main lymphoid and myeloid cell subsets known to be enriched in a developing tumor [45]. EpCAM was used to segment the tissue into epithelial and stroma compartments. It’s interesting to note that the only cell subset that was significantly enriched in the infiltrate of both, epithelium and stroma compartments of colon tissue from CNF1 + DSS-treated animals, as compared to DSS-treated animals, was CD11b + Ly6G+, i.e., neutrophils (Fig. 6H-I), as also evidenced by the H&E staining (Fig. 6C; Table 1). No difference in the abundance of the lymphoid and other myeloid subsets analysed was observed between the two groups (Supplementary Figs. 4–5). However, a moderate increase in Foxp3 + and PD1 + cells, putative regulatory T cells (T-regs), was observed in the stromal compartment of tissue sections from CNF1 + DSS compared to DSS, even though the result did not reach statistical significance (Supplementary Fig. 5). Overall, these data demonstrate the causal association between CNF1 and CRC in an IBD animal model.

### CNF1-induced tumorigenesis is associated with alterations in fecal microbiota composition

**Fig. 7:**
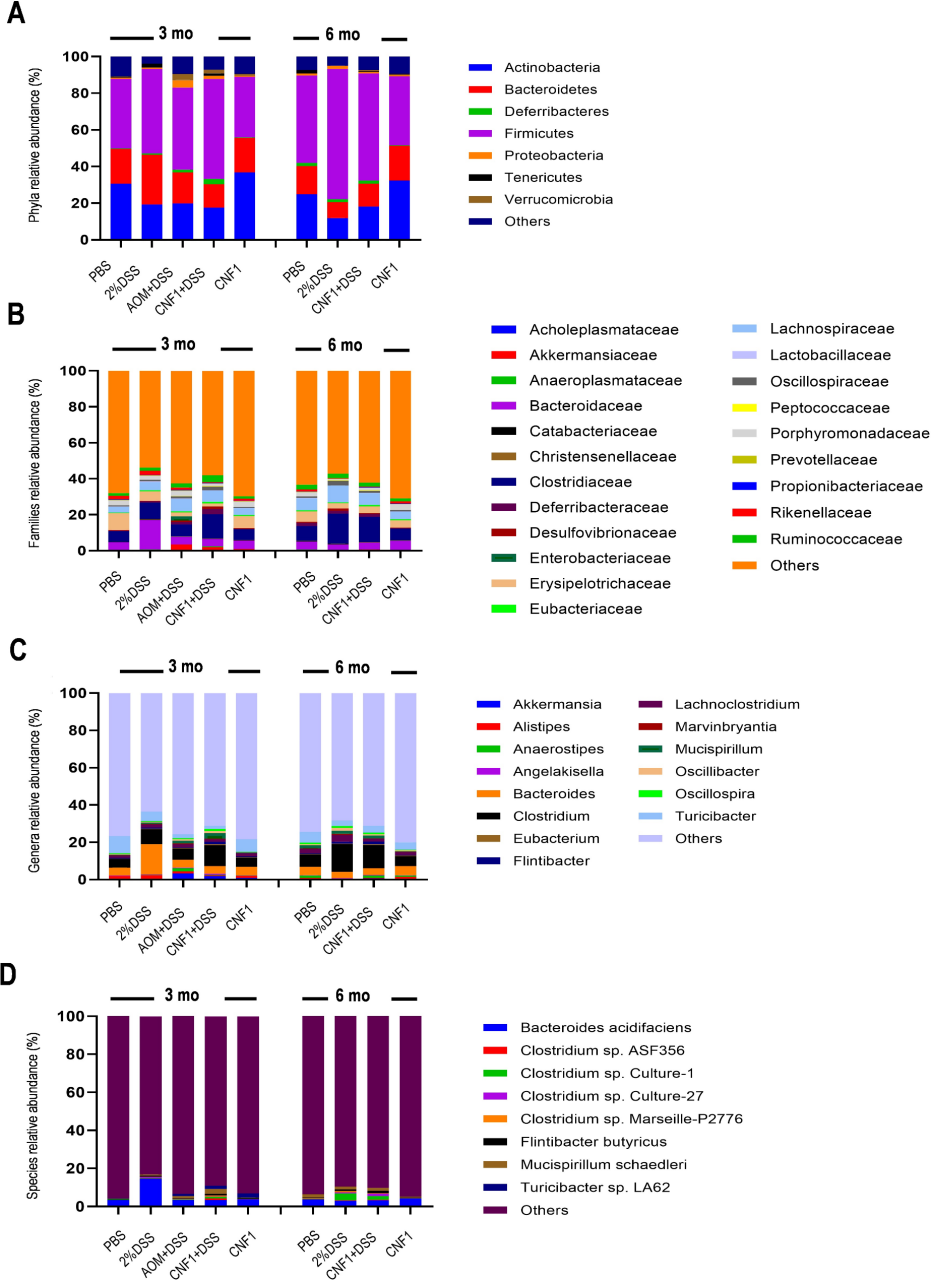
CNF1-induced modification of gut microbiota. Stacked barplots showing the mean relative abundance of gut bacterial phyla (**A**), families (**B**), genera (**C**) and species (**D**) 3 and 6 months after treatment. The “Others” category includes unknown bacteria and all other microbes whose mean relative abundance is less than 0.1% at phylum and family level, and 0.5% at genus and species level, respectively

Since microbiota composition is a key environmental factor in intestinal carcinogenesis, we performed 16 S rRNA gene sequencing using faecal pellets collected from all groups of treatment to identify changes in microbiota composition associated with CRC. No significant difference among the groups was observed as for bacterial richness and diversity indices (data not shown). On the other hand, several statistically significant changes were observed at all taxonomic levels when comparing the groups with each other. Bacterial relative abundance at the phylum, family, genus and species levels are shown in Fig. 7A-D. At three months of the experimental protocol, as compared to PBS control, CNF1 group exhibited an increase in the phylum *Verrucomicrobia* (0.959% vs. 0.146%) and its related family *Akkermansiaceae* (0.952% vs. 0.141%) while a decrease in *Tenericutes* (0.052% vs. 0.556%) and its cognate family *Anaeroplasmataceae* (0.010% vs. 0.165%) was evident. The genus *Akkermansia* (0.950% vs. 0.140%) and the species *Ruminococcus flavefaciens* (0.107% vs. 0.006%) were also significantly enriched. When comparing the CNF1 + DSS group with DSS alone, a greater number of significant variations in the gut microbial profile were observed. At the phylum level, again *Verrucomicrobia* (1.941% vs. 0.014%) were increased together with *Deferribacteres* (2.926% vs. 0.726%), whereas *Bacteroidetes* were found depleted (12.750% vs. 27.119%). Among the families, *Akkermansiaceae* (1.925% vs. 0.014%), *Deferribacteraceae* (2.884% vs. 0.726%), *Desulfovibrionaceae* (1.276% vs. 0.320%), *Oscillospiraceae* (2.150% vs. 0.831%), *Peptococcaceae* (0.218% vs. 0.093%) and *Rumonicoccaceae* (3.741% vs. 1.700%) were more abundant, while *Bacteroidaceae* (4.225% vs. 16.413%), *Lactobacillaceae* (0.095% vs. 0.406%), *Rikenellaceae* (0.520% vs. 2.421%) and *Erysipelotrichaceae* (1.842% vs. 5.163%) were decreased in the same comparison. Among the genera, *Hespellia* (0.319% vs. 0.000%), *Anaerosporobacter* (0.202% vs. 0.000%), *Akkermansia* (1.909% vs. 0.012%), *Butyricicoccus* (0.439% vs. 0.060%), *Anaerotruncus* (0.211% vs. 0.044%), *Robinsoniella* (0.095% vs. 0.023%), *Mucispirillum* (2.864% vs. 0.724%), *Anaerostipes* (0.164% vs. 0.044%), *Oscillospira* (1.066% vs. 0.365%), *Angelakisella* (0.637% vs. 0.230%), *Eubacterium* (0.630% vs. 0.248%), *Oscillibacter* (1.190% vs. 0.473%) and *Flintibacter* (1.131% vs. 0.532%) were up-represented whereas *Alistipes* (0.482% vs. 2.386%), *Lactobacillus* (0.084% vs. 0.402%), *Bacteroides* (4.114% vs. 16.302%), *Parabacteroides* (0.096% vs. 0.325%) and *Turicibacter* (1.746% vs. 5.066%) were down-represented. Finally, an increased abundance of the species *Turicibacter sp.* LA62 (1.656% vs. 0.021%), *Oscillibacter valericigenes* (0.074% vs. 0.004%) and *Mucispirillum schaedleri* (2.617% vs. 0.063%) and a decreased abundance of *Bacteroides acidifaciens* (3.478% vs. 14.702%) was recorded. The gut microbial profiles of the animal groups were analysed also later at the end of six months of the experimental protocol. At the phylum level, when CNF1 group was compared to control, the same significant changes as at 3 months were observed, with *Verrucomicrobia* enriched (0.418% vs. 0.049%) and *Tenericutes* (0.215% vs. 1.896%) depleted. Going down in the taxonomic scale, the families *Enterobacteriaceae* (0.109% vs. 0.03%) and *Akkermansiaceae* (0.417% vs. 0.048%), the genera *Escherichia* (0.053% vs. 0.000%) and *Akkermansia* (0.416% vs. 0.047%), and the species *Escherichia coli* (0.052% vs. 0.000%) and *Ruminococcus flavefaciens* (0.078% vs. 0.016%) were found up-represented while the families *Anaeroplasmataceae* (0.042% vs. 0.473%), *Acholeplasmataceae* (0.013% vs. 0.080%) and *Deferribacteraceae* (0.266% vs. 1.735%), the genera *Anaeroplasma* (0.009% vs. 0.105%) and *Mucispirillum* (0.265% vs. 1.733%) and the species *Mucispirillum schaedleri* (0.248% vs. 1.601%) were found down-represented. When comparing the CNF1 + DSS group with DSS, instead, the phyla *Verrucomicrobia* (0.602% vs. 0.001%) and *Tenericutes* (0.288% vs. 0.025%), the families *Akkermansiaceae* (0.601% vs. 0.001%) and *Anaeroplasmataceae* (0.073% vs. 0.007%), the genera *Akkermansia* (0.599% vs. 0.001%), *Hespellia* (0.177% vs. 0.001%), *Escherichia* (0.036% vs. 0.000%), *Anaerosporobacter* (0.384% vs. 0.004%), *Anaerostipes* (1.252% vs. 0.212%) and *Butyricicoccus* (0.292% vs. 0.085%), and the species *Escherichia coli* (0.035% vs. 0.000%) were found increased whereas only the genus *Marvinbryantia* (0.048% vs. 0.640%) and the species *Lactobacillus reuteri* (0.000% vs. 0.033%) were found decreased.

## Discussion

The concept of bacteria-host interactions playing a significant role in CRC development is being reinforced by the notion that not only specific microorganisms, but also their virulence factors or metabolites, can induce human carcinogenesis [46]. In this scenario, CNF1 from *E. coli* has been suggested to be involved in the pathogenesis of CRC given its impact on Rho GTPases and the consequent interference with multiple key cellular pathways linked to neoplastic transformation [47, 48]. Furthermore, this toxin gene was found to be associated with colorectal neoplastic tissue in CRC patients [16] while *E. coli* toxins as a whole are over-represented in adenocarcinomas from patients undergoing colonoscopy [15]. Despite the abundance of evidence currently available, a definitive causal link between CNF1 and colorectal carcinogenesis remains elusive. Our results indicate, for the first time to the best of our knowledge, that CNF1 induces genetic instability in vitro and that repeated exposure of inflamed colon tissue to CNF1 causes colorectal adenomas in vivo.

Colorectal carcinogenesis is a multi-step process during which cells accumulate alterations in genes and proteins resulting in the gradual transformation into neoplastic cells [49]. The process is significantly influenced by continuous oxidative stress and chronic inflammation. The ability of CNF1 to stimulate the production of ROS and the release of pro-inflammatory cytokines through the activation of Rho-GTPases had previously been observed in epithelial cells in vitro [50]. In the present study, we show that CNF1 not only induces an increase in oxidative stress level in non-neoplastic intestinal epithelial cells, but also causes the accumulation of chromatid breaks and increases the number of polyploid cells, which are pre-requisites of neoplastic transformation [51]. Indeed, it has been already demonstrated that certain secreted bacterial toxins play an active role in colorectal carcinogenesis. This is the case for colibactin from *E. coli* and BFT from *B. fragilis*, as well as FadA from *Fusobacterium nucleatum* and CDT produced by several bacterial species [46, 52]. Regardless of their mechanism of action, these toxins increase the risk of cancer development via DNA damage, whether directly or indirectly induced. In fact, in addition to colibactin, which generates inter-strand crosslinks [53, 54] and CDT, which possesses a DNAse activity [55], recent studies have shown that BFT and FadA, historically regarded as toxins inducing CRC through the manipulation of specific cell signalling pathways, are also able to damage DNA through the induction of oxidative stress [56, 57]. Based on our results, we can conclude that CNF1, which was putatively included in the group of toxins causing cancer through subversion of cell signalling, utilize the signals downstream Rho GTPases activation to damage DNA as well and induce genomic instability though both ROS-dependent and ROS-independent mechanisms. In fact, our experiments correlating oxidative stress with DNA damage showed that pre-treatment of epithelial cells with the antioxidant compound NAC significantly decreased γH2AX positive nuclei, while chromatid breaks were not significantly affected. These results suggest that the observed ROS-dependent phosphorylation of H2AX is mainly due to the accumulation of SSB, while the DSB observed in metaphase may follow alternative pathways that are independent of ROS activity. This conclusion is also supported by the activation of ATR/CHK1 and by the low number of 53BP1 and γH2AX foci co-localizations observed in CNF1-treated cultures, with respect to the total number of γH2AX foci, being 53BP1 a specific marker of DSB [58]. Conversely, the observed reduction of polyploid cells during metaphase in CNF1-exposed epithelial cells pre-treated with NAC, may indicate a ROS-mediated protein damage. Constitutive activation of Rho-GTPases results in the actin cytoskeleton reorganization [59]that, in turn, can hinder cytokinesis, leading to the formation of binucleated cells that, upon division, will generate polyploid cells. The decrease in polyploidy induced by pre-treatment with NAC indicates that the antioxidant may affect polyploidy via downstream ROS production. It is worth noting that ROS are numerous and diverse, and may react with each other to form new reactive products. For example, peroxynitrite, a product of the reaction between superoxide anion and nitric oxide, is responsible for post-translational modification of proteins [60]. Future research will focus on the identification of the specific ROS type(s) produced as a consequence of CNF1 encounter, in order to identify the potential intracellular targets affected by the oxidative action of the toxin and gain insight on the exact molecular mechanisms of CNF1 genotoxic effect.

Numerous studies show that DSBs and DDR are increased in precancerous lesions. While the precise molecular mechanisms of CNF1-induced genotoxicity remain to be fully elucidated, our findings indicate that repeated intrarectal administration of CNF1 alone induces 53BP1-positive staining and dysplastic ACF, which are the earliest developing precursors of epithelial neoplasms [61], in the colonic tissue. Most importantly, in a mouse model of IBD, a known risk-factor for CRC, intrarectal CNF1 administration induces a significantly higher count of dysplastic ACF and 53BP1 in comparison to controls, and the formation of focal adenomas, diffuse GIN and squamous metaplasias. These pathological alterations are early morphologic changes of colon tissue that are commonly found in colons of mice and rats treated with a carcinogen [35].

Although the continuous acquisition of oncogenic mutations drives neoplastic transformation, the type of immune microenvironment plays an active role in defining tumor fate. The inflammatory process triggered by a pathogen or by tissue damage is controlled by a complicated balance of pro- and anti-inflammatory cytokines. Our results show that the genes encoding for the main pro-inflammatory cytokines, such as IL-1β, IL-6, also known to be involved in the pathogenesis of DSS-induced experimental colitis [62], and for IL-8, which is a potent neutrophil chemoattractant, are also upregulated by CNF1. Simultaneously, a switch towards anti-inflammatory cytokine production (i.e. IL-10) and pro-inflammatory cytokine down-regulation (i.e. TNF-α) is observed in CNF1-treated mice, suggesting that the toxin may accelerate tissue repair and inflammation resolution supporting the hyperproliferation of aberrant cells. In addition, since in these animals the upregulation of IL-10, is not counterbalanced by the induction of IFNγ, we can speculate that CNF1 may also favour immune evasion of genomic instable aberrant cells, thus accelerating tumorigenesis.

In this respect, the observed increased neutrophilic infiltration in colon tissue of CNF1 + DSS-treated animals may be supportive of the carcinogenic process. In fact, it has been previously shown that, by secreting IL-1β, neutrophils infiltrating the colon tissue participate in transforming from IBD to CAC [63]. Among the proposed tumorigenic mechanisms of neutrophils, there is the reduction of CD8 + T cell infiltration and function through the secretion of arginase-1, and the CCL17-mediated recruitment of T-regs [64–66]. In this regard, we may speculate that the moderate increase in Foxp3 and PD-1 expression in colon tissue of CNF1 + DSS-treated mice may be an early consequence of the immune-suppressive and tumor-promoting modulation exerted by IL-10 and neutrophils.

It is known that intestinal dysbiosis contributes to alterations of gut permeability, which allow the passage of microbes and inflammatory substances and, as such, is implicated in colorectal carcinogenesis [67]. In this regard, a key role is played by the mucus layer. The observed upregulation of *Muc2* gene expression in CNF1- and CNF1 + DSS-treated animals may reflect an altered production of MUC-2 by goblet cells to protect the gut mucosa from infection. However, under repeated or continuous bacterial stimulation, prolonged overexpression of *Muc2* in goblet cells may lead to a dysfunctional MUC2 production, with misfolded proteins accumulating in the ER, to induce ER stress and initiate the unfolded protein response (UPR) [68], a signalling pathway contributing to cellular transformation [69]. Furthermore, our results indicate that CNF1 itself can alter the epithelial TJ distribution in vitro through its Rho GTPases-dependent activity and that the addition of an inflammatory supernatant strongly accelerates this effect. Nevertheless, we believe that an increase in intestinal permeability is not sufficient per se to induce neoplastic transformation in colon tissue. Indeed, in vivo, animals treated with both CNF1 + DSS versus DSS alone exhibited comparable degrees of gut permeability. However, only those treated with the former developed colorectal tumors allowing the conclusion that, in our system, inflammation was necessary, but not sufficient to induce CRC. In addition to the already discussed genotoxic effects, data from fecal microbiota analysis indicate that CNF1 alters the composition of the gut microbiota, creating a CRC-permissive environment by promoting the growth of pro-inflammatory and pro-tumorigenic bacteria. A common feature of the experimental groups receiving CNF1, when compared to their respective counterparts, at all time-point, is a significant increase in the phylum *Verrucomicrobia* and, downstream, in the family *Akkermansiaceae* and the genus *Akkermansia*. In particular, the increase was attributable to an unclassified species within the genus *Akkermansia*, whose family members’ role in pathophysiology of the gut is quite controversial [70–72]. In mice treated with CNF1 + DSS, an enrichment in some detrimental bacteria and a decrease in some beneficial microbes was observed after three months in comparison to DSS alone. Among the enriched bacteria, *Deferribacteres*, are considered as a negative prognostic factor for CRC [73] and its cognate species *Mucispirillum schaedleri*, has been associated with intestinal inflammation [74]. *Desulfovibrionaceae*, a family known to contribute to the development and progression of CRC [75], were also enriched in CNF1 + DSS vs. DSS. In contrast, *Lactobacillaceae* and *Lactobacillus*, which are generally regarded as beneficial bacteria capable of strengthening the intestinal barrier, preventing pathogen colonization and enhancing antitumor immunity [76–78] were found reduced in our model. It is interesting to note that the analyses of the microbial profiles in faecal samples collected at six months revealed a significant enrichment of *E. coli* in the CNF1 and CNF1 + DSS groups compared to their controls (PBS and DSS), suggesting that long-term exposure to the *E. coli*–derived toxin promotes *E. coli* growth in the gut. This observation is consistent with a recent study demonstrating that CNF1 confers high invasive capacities of epithelial cells to *E. coli* and acts as an intestinal colonization factor during competition in the gastrointestinal tract [79]. In addition, some studies have reported higher levels of colonization by mucosa-associated *E. coli* in patients with CRC compared to healthy patients [80, 81]. Therefore, we hypothesize that CNF1 may further contribute to colorectal tumorigenesis by conferring a competitive advantage to pathogenic *E. coli* strains. In addition, the depletion of *L. reuteri* was observed in mice treated with CNF1 + DSS, as compared to the DSS group. This bacterium and its major metabolite reuterin have been found to be decreased in CRC in both mice and humans, suggesting a cancer-suppressive function [82]. Thus, we can conclude that CNF1 can also modulate gut microbiota and intestinal permeability.

Pathogenic *E. coli* are the most common gram-negative bacterial pathogen in humans. They can cause a variety of extraintestinal diseases ranging from urinary tract infections (UTIs), such as bladder or kidney infections, to severe bacteremia and septic shock [83]. In 2017, the World Health Organization recognized sepsis as a global health priority [84]. Antimicrobial treatment is usually the first-line of defence against these pathogens; however multiple antimicrobial resistance is emerging as a major obstacle for pathogenic *E. coli* eradication. In recent years, significant progress has been made toward the development of vaccine against defined pathogenic *E. coli* strains. This vaccine showed an acceptable safety profile and was able to elicit a robust immune response in adults (≥ 60 years) with a history of UTIs, a population known to have an increased risk for invasive *E. coli* disease [85]. Another vaccine with a similar composition is presently being evaluated in a phase III clinical trial (NCT04899336). If our results demonstrating the genotoxic effect of CNF1 and its tumorigenic potential could be extended to humans, we would envisage the possibility that a targeted reduction of pathogenic *E. coli* via vaccination could prevent or delay tumor development in patients with an increased risk for CRC, such as those with IBD.

## Conclusions

In summary, our research reveals that CNF1 from *E. coli* plays an active role in colorectal carcinogenesis. In vivo, repeated exposure of the colonic mucosa to CNF1 induces the formation of dysplastic ACF and, in an IBD mouse model, stimulates the formation of colorectal adenomas. These effects are accompanied by the increased neutrophilic infiltration into the colonic tissue, by a mixed pro-inflammatory and anti-inflammatory cytokine milieu and by the pro-tumoral modulation of the fecal microbiota. Mechanistically, CNF1 induces the production of reactive oxidizing species, genotoxicity and gut permeability alteration. Altogether, these findings not only add new knowledge to the contribution of bacterial toxins to CRC, but also pave the way to the implementation of current screening programs and preventive strategies.

## Electronic supplementary material

Below is the link to the electronic supplementary material.

Additional file 1: Supplementary Table 1: List of antibodies

Additional file 2: Supplementary Table 2: List of antibodies, Opal and retrival buffers used for Multiplex IF immunostaining

Additional file 3: Supplementary Table 3: List of primers used for real-time PCR

Additional file 4: Supplementary Table 4: Scoring parameters for colonoscopy

Additional file 5: Supplementary Figure 1: CNF1 effects on HPCEC cells. Supplementary Figure 2: CNF1-induced DDR in IEC-6 and HPCEC cells. Supplementary Figure 3: Body weight and gut permeability monitoring. Supplementary Figure 4: Six-channel multiplexed IF imaging of myeloid infiltrates in 2%DSS vs CNF1+DSS treated animals. Supplementary Figure 5: Six-channel multiplexed staining of T lymphocyte infiltrates in 2%DSS vs CNF1+DSS− treated animals

## Data Availability

All data generated in this study are available within the article and its Supplementary information files. FASTQ files containing 16 S rRNA gene sequencing raw data were deposited in ArrayExpress under the accession code E-MTAB-14592. All other raw data are available upon request from the corresponding author.

## Abbreviations

53BP1: P53-binding protein 1
AOM: Azoxymethane
ACF: Aberrant crypt foci
CAC: Colitis-associated colorectal
CM: Conditioned medium
CNF1: Cytotoxic necrotizing factor-1
CP^•^: 3-carboxy-proxyl radical
CRC: Colorectal cancer
DAPI: 4’,6-diamidino-2-phenylindole
DDR: DNA damage response
DMEM: Dulbecco’s modified eagle’s medium
DSB: Double-strand break
DSS: Dextran sulfate sodium
EPR: Electron paramagnetic resonance
FFPE: Formalin-fixed paraffin-embedded
GIN: Gastrointestinal intraepithelial neoplasia
γH2AX: Histone H2AX
H&E: Haematoxylin and eosin
HPCECs: Human primary colonic epithelial cells
IBD: Inflammatory bowel disease
i.r.: Intrarectal
MFI: Mean fluorescence intensity
NAC: N-acetyl cysteine
NEAA: Non-essential amino acids
PFA: Paraformaldehyde
pRB: Retinoblastoma protein
ROS: Reactive oxidizing species
RT: Room temperature
SEM: Scanning electron microscopy
TEER: Trans-epithelial electrical resistance
TEM: Transmission electron microscopy
TJs: Tight junctions
T-regs: Regulatory T cells
UTIs: Urinary tract infections
WB: Western blot
