# Supplementary material for: E. Coli cytotoxic necrotizing factor-1 promotes colorectal carcinogenesis by causing oxidative stress, DNA damage and intestinal permeability alteration
Source: J Exp Clin Cancer Res. 2025 Jan 29;44:29. doi: 10.1186/s13046-024-03271-w (PMC11776187; doi:10.1186/s13046-024-03271-w)
Supplement: Supplementary file 1 — Additional file 1: Supplementary Table 1: List of antibodies [file 13046_2024_3271_MOESM1_ESM.docx]

**Supplementary Table 1.** List of antibodies used for Fluorescence Microscopy (FM), Western Blot (WB), Immunohistochemistry (IHC), and Confocal laser scanning microscopy (CLSM) stainings.

| Antibody (clone) | Assay | Supplier (catalog n°) | Dilution factor |
| --- | --- | --- | --- |
| γH2AX (S139) | FM | Cell Signaling Technology (#2577S) | 1:400 |
| γH2AX (3F2) | WB | Abcam (#ab22551) | 1:6000 |
| pRB (G3-245) | WB | BD Biosciences (#AB_395259) | 1:500 |
| 53BP1(Polyclonal) | FM/IHC | Thermo Fisher Scientific (#PA1-16566) | 1:200/ 1:300 |
| p21(SX118) | /WB | BD Biosciences (#558430) | 1:500 |
| alpha-tubulin (polyclonal) | WB | Cell Signaling Technology (#2144) | 1:1000 |
| ZO-1 (1/ZO-1) | CLSM | BD Biosciences (#610967) | 1:50 |
| Alexa Fluor-488 F(ab)2 fragments of goat anti-mouse IgG (Polyclonal) | FM/CLSM | Thermo Fisher Scientific (#A-11017) | 1:200 |
| Alexa Fluor-594 F(ab)2 fragments of goat anti-rabbit IgG (Polyclonal) | FM/CLSM | Thermo Fisher Scientific (#A-11072) | 1:200 |
| DAPI | FM/CLSM | Thermo Fisher Scientific (#D1306) | 1:300 |
| Alexa Fluor-594 phalloidin (Polyclonal) | FM/CLSM | Thermo Fisher Scientific (#A12381) | 1:50 |
| HRP-conjugated goat anti-rabbit antibody | IHC | Abcam (#ab7090) | 1:300 |
| HSC70 (W27) | WB | Santa Cruz (#SC-7298) | 1:500 |
| Cyclin D1 (A-12) | WB | Santa Cruz (#SC-8396) | 1:500 |
| p-53 (DO-7) | WB | BD Biosciences (#AB-554247) | 1:1000 |
| p-p53 (polyclonal) | WB | Invitrogen (#PA5-104742) | 1:1000 |
| Chk1 (DCS-310) | WB | Santa cruz (#SC-8408) | 1:1000 |
| phChk1 (polyclonal) | WB | Cell Signaling Technology (#5345) | 1:1000 |
