# Supplementary material for: E. Coli cytotoxic necrotizing factor-1 promotes colorectal carcinogenesis by causing oxidative stress, DNA damage and intestinal permeability alteration
Source: J Exp Clin Cancer Res. 2025 Jan 29;44:29. doi: 10.1186/s13046-024-03271-w (PMC11776187; doi:10.1186/s13046-024-03271-w)
Supplement: Supplementary file 2 — Additional file 2: Supplementary Table 2: List of antibodies, Opal and retrival buffers used for Multiplex IF immunostaining [file 13046_2024_3271_MOESM2_ESM.docx]

**Supplementary Table 2.** List of antibodies, Opal and retrival buffers used for Multiplex IF immunostaining.

**a) Myeloid cells mix**

| Order | Antibody | Supplier | Clone | Catalog | Diluition Factor | Opal Pairing | Retrival and Incubation Time |
| --- | --- | --- | --- | --- | --- | --- | --- |
| 1 | LY6G | Abcam | EPR22909-135 | ab238132 | 1:500 | 520 | ER2 30’ |
| 2 | CD68 | CST | E3O7V | 97778 | 1:600 | 570 | ER2 30’ |
| 3 | CD11b | Abcam | EPR1344 | ab133357 | 1:3000 | 480 | ER1 30’ |
| 4 | PD-L1 | CST | D5V3B | 64988 | 1:100 | 690 | ER2 30’ |
| 5 | EpCAM | CST | E6V8Y | 93790 | 1:200 | 780 | ER2 30’ |

**b) Lymphocytes mix**

| Order | Antibody | Supplier | Clone | Catalog | Diluition Facor | Opal Pairing | Retrival and Incubation Time |
| --- | --- | --- | --- | --- | --- | --- | --- |
| 1 | FOXP3 | CST | D6O8R | 12653 | 1:800 | 520 | ER2 30’ |
| 2 | CD4 | Abcam | RM1013 | AB288724 | 1:1000 | 579 | ER2 30’ |
| 3 | CD8 | Abcam | EPR21769 | AB217344 | 1:2000 | 620 | ER2 30’ |
| 4 | PD-1 | CST | D7D5W | 84651 | 1:150 | 480 | ER2 30’ |
| 6 | EpCAM | CST | E6V8Y | 93790 | 1:200 | 780 | ER2 30’ |
