# Supplementary material for: E. Coli cytotoxic necrotizing factor-1 promotes colorectal carcinogenesis by causing oxidative stress, DNA damage and intestinal permeability alteration
Source: J Exp Clin Cancer Res. 2025 Jan 29;44:29. doi: 10.1186/s13046-024-03271-w (PMC11776187; doi:10.1186/s13046-024-03271-w)
Supplement: Supplementary file 3 — Additional file 3: Supplementary Table 3: List of primers used for real-time PCR [file 13046_2024_3271_MOESM3_ESM.docx]

| **Gene name** | **forward (FW) 5'-3'** | **reverse (RV) 5'-3'** |
| --- | --- | --- |
| *Rplp0* | AGATTCGGGATATGCTGTTGGC | TCGGGTCCTAGACCAGTGTTC |
| *Il1β* | TTC GTG AAT GAG CAG ACA GC | CCA TGG TTT CTT GTG ACC CT |
| *Il6* | GTCCTTCCTACCCCAATTTCCA | CGCACTAGGTTTGCCGAGTA |
| *Il8* | ACTCAAGAATGGTCGCGAGG | GTGCCATCAGAGCAGTCTGT |
| *Il10* | TGGGTTGCCAAGCCTTATCG | TTCAGCTTCTCACCCAGGGA |
| *Tnfα* | GATCGGTCCCCAAAGGGATG | TTTGCTACGACGTGGGCTAC |
| *Ifnγ* | ACGGCACAGTCATTGAAAGC | TTTCATGTCACCATCCTTTTGCC |
| *Muc-2* | GATGGCACCTACCTCGTTGT | GTCCTGGCACTTGTTGGAAT |

**Supplementary Table 3.**Primers list.
