# Supplementary material for: E. Coli cytotoxic necrotizing factor-1 promotes colorectal carcinogenesis by causing oxidative stress, DNA damage and intestinal permeability alteration
Source: J Exp Clin Cancer Res. 2025 Jan 29;44:29. doi: 10.1186/s13046-024-03271-w (PMC11776187; doi:10.1186/s13046-024-03271-w)
Supplement: Supplementary file 4 — Additional file 4: Supplementary Table 4: Scoring parameters for colonoscopy [file 13046_2024_3271_MOESM4_ESM.docx]

**Supplementary Table 3.** Scoring parameters for colonoscopy

| SCORE | 0 | 1 | 2 | 3 |
| --- | --- | --- | --- | --- |
| **Vascular pattern** | Normal | Partial loss | Total loss | - |
| **Granularity** | Smooth | Mild | Moderate | Severe |
| **Thickening of colon mucosa** | Transparent | Mild | Moderate | Thickened |
| **Fibrin visible** | None | Little | Marked | Extreme |
| **Spontaneous bleeding** | None | Mild | Moderate | Severe |
| **Stool consistency** | Normal | Soft | Loose | - |
| **Erosions/ulcerations** | None | Erosion | Superficial ulceration | Deep ulceration |
| **Tumor lesions** | None | Sporadic | Diffuse | - |
