# Supplementary material for: E. Coli cytotoxic necrotizing factor-1 promotes colorectal carcinogenesis by causing oxidative stress, DNA damage and intestinal permeability alteration
Source: J Exp Clin Cancer Res. 2025 Jan 29;44:29. doi: 10.1186/s13046-024-03271-w (PMC11776187; doi:10.1186/s13046-024-03271-w)
Supplement: Supplementary file 5 — Additional file 5: Supplementary Figure 1: CNF1 effects on HPCEC cells. Supplementary Figure 2: CNF1-induced DDR in IEC-6 and HPCEC cells. Supplementary Figure 3: Body weight and gut permeability monitoring. Supplementary Figure 4: Six-channel multiplexed IF imaging of myeloid infiltrates in 2%DSS vs CNF1+DSS treated animals. Supplementary Figure 5: Six-channel multiplexed staining of T lymphocyte infiltrates in 2%DSS vs CNF1+DSS− treated animals [file 13046_2024_3271_MOESM5_ESM.pdf]

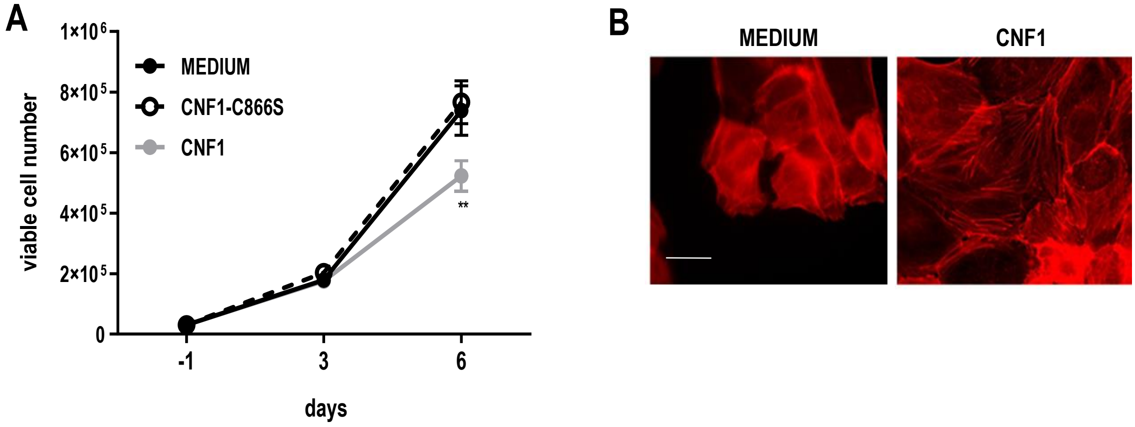

Supplementary Figure 1. CNF1 effects on HPCEC cells. (A) Trypan blue exclusion count of HPCEC cells after 3 and 6 days of culture in the presence of CNF1 or CNF1-C866S (25 pM, N=3). (**$p<0.01$ vs CNF1-C866S and PBS). **(B)** Representative immunofluorescence micrographs of phalloidin staining of HPCEC cells treated with 25 pM CNF1 for 24 h or untreated controls. Scale bar:10 μm**.**

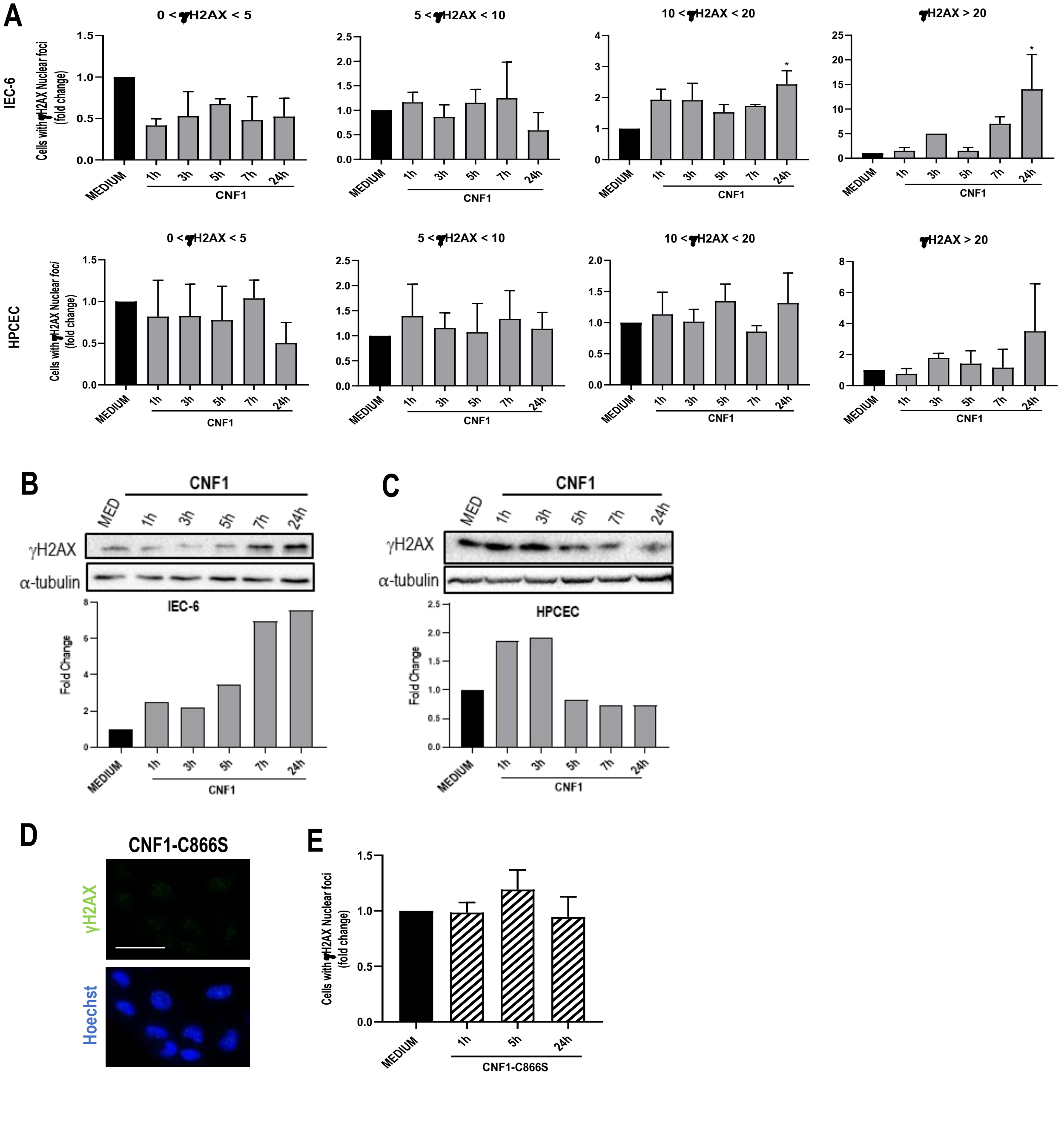

Supplementary Figure 2. CNF1-induced DDR in IEC-6 and HPCEC cells. (A) Classes of nuclear *foci* number of γH2AX in IEC-6 and HPCEC cells. (*p<0.05). **(B-C)** Expression level of γH2AX in IEC-6 and HPCEC cells by western blot. Representative micrographs and relative densitometry bar plots showing the fold change of γH2AX in IEC-6 and HPCEC cells at each indicated time-point as compared to untreated cells. One representative experiment out of three with similar results is shown. **(D)** Representative fluorescence micrographs of IEC-6 cells stained with anti-γH2AX (green). Nuclei were counterstained with Hoechst (blue). Scale bar: 10 µm. **(E)** Bar plot showing the fold change of γH2AX-positive nuclei at different time points following exposure to 25 pM CNF1-C866S mutant toxin.

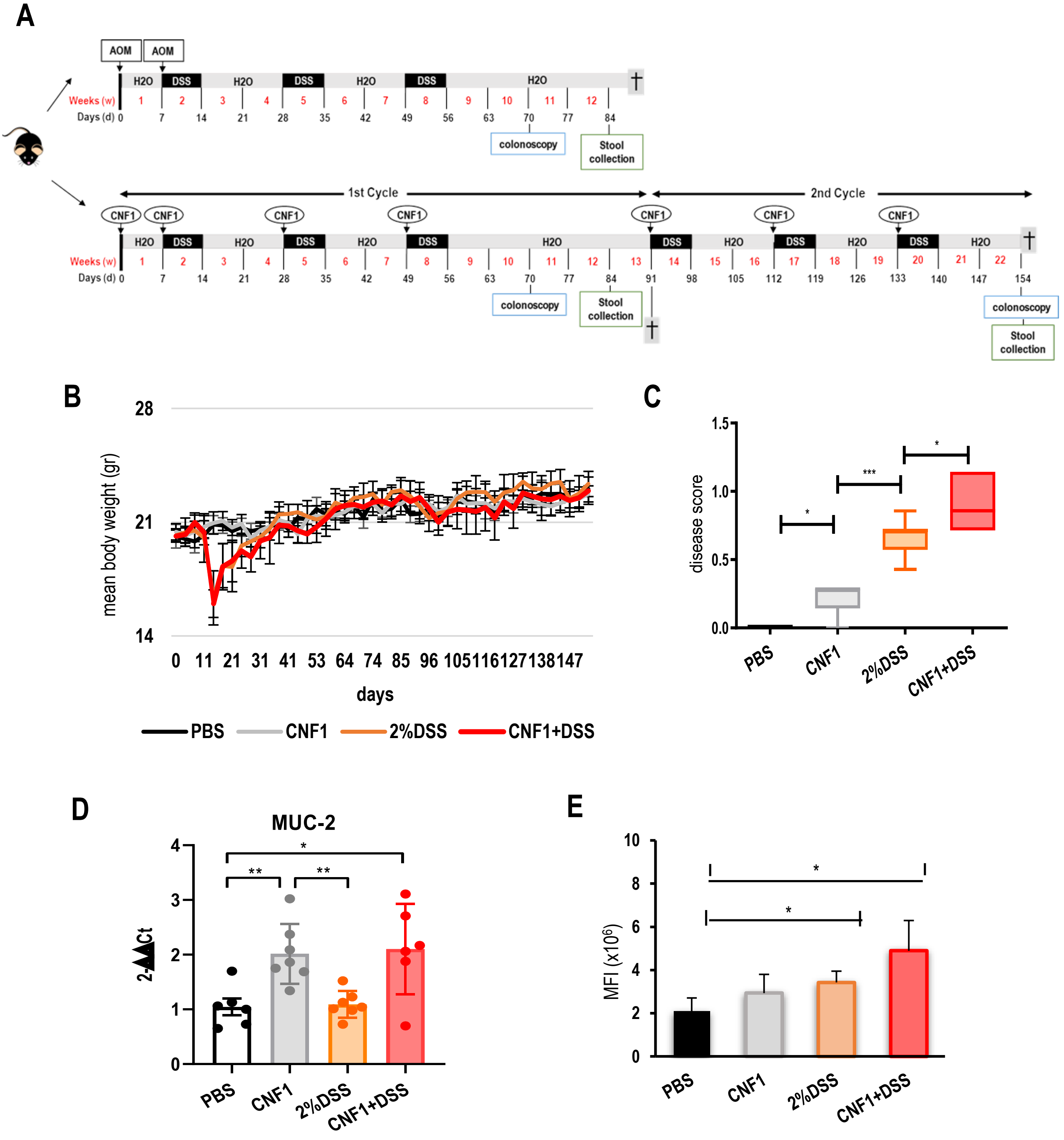

Supplementary Figure 3. Body weight and disease monitoring. (A) Treatment schedule of the AOM/DSS model and of the experimental model in which AOM/DSS was replaced by three cycles of i.r CNF1 administration. **(B)** Mean body weight of mice (N=10) in each experimental group of the six cycles of i.r CNF1 administration. One representative experiment out of two with similar results is shown. **(C)** Boxplot depicting the disease score generated at colonoscopy. Center lines show the medians; box limits indicate the 25th and 75th percentiles; whiskers represent Min and Max values. (*p<0.05; ***p<0.001). **(D)** Gene expression analysis of MUC-2 by real-time PCR in colon tissue. **(E)** Dextran-FITC Fluorescence intensity in plasma samples from the indicated treatment groups (N=5 per group). (*p<0.05).

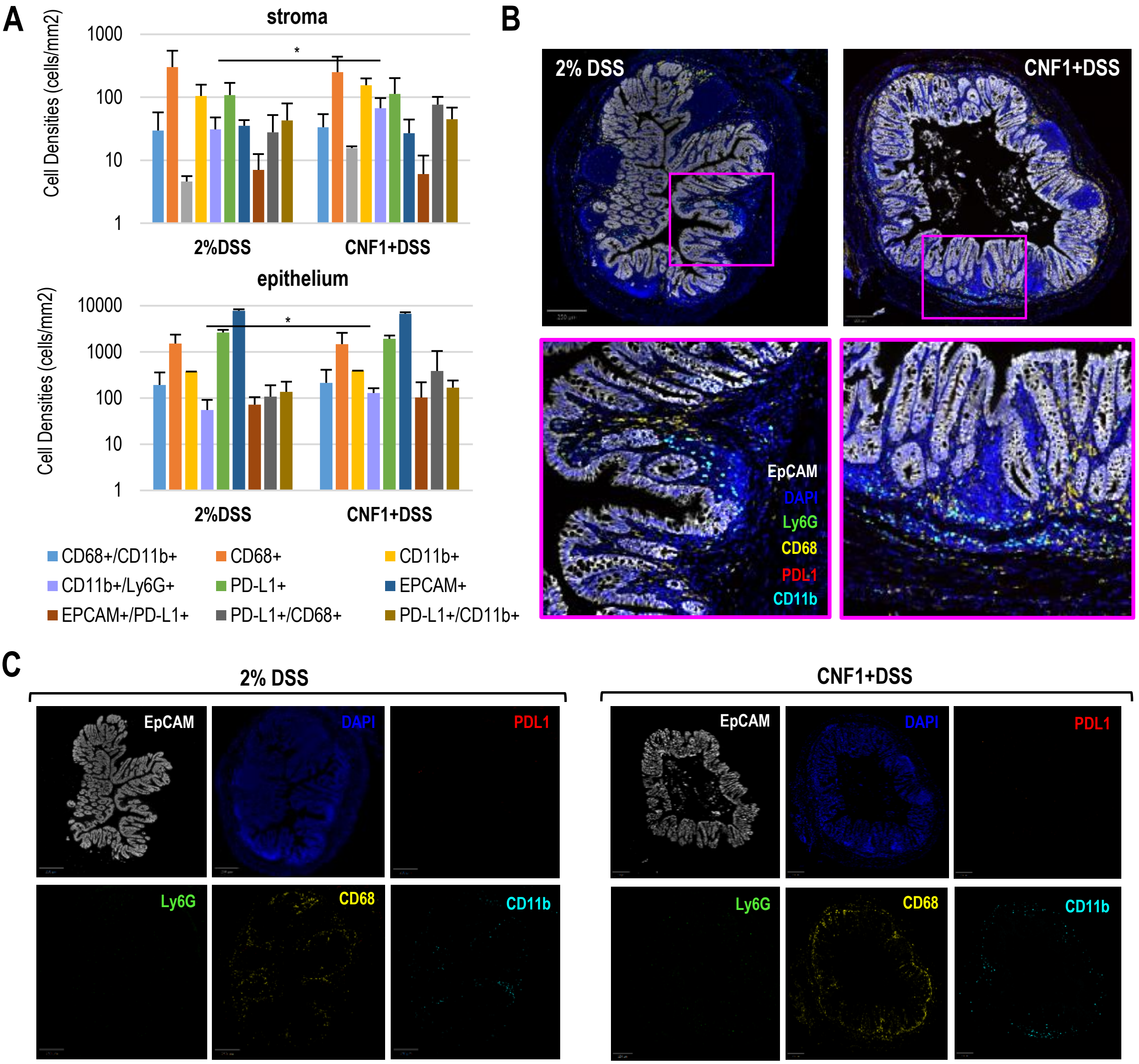

Supplementary Figure 4. Six-channel multiplexed IF imaging of myeloid infiltrates in 2% DSS vs CNF1+DSS treated animals. (A) Bar plots showing the density of the indicated marker or marker combination in the epithelium or stroma compartments of colon tissue. (*$p<0.05$). **(B)** Representative images of colons from the indicated treatment groups (N=7 per group) multiple stained for EpCAM (white),CD11b (light blue) Ly6G (green), CD68 (yellow) PDL1 (red), and DAPI (blue). **(C)** Representative images of each single channel.

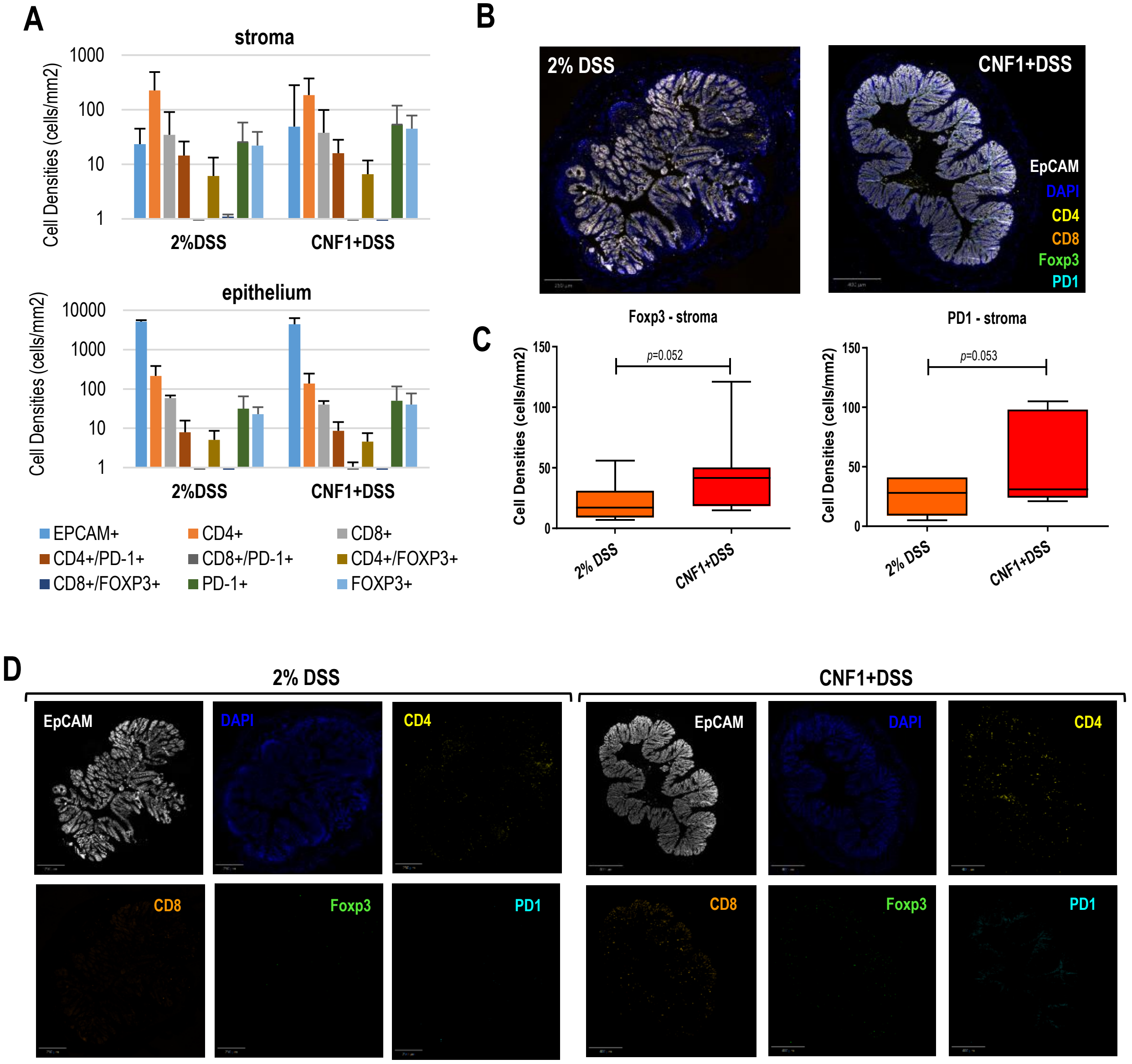

Supplementary Figure 5. Six-channel multiplexed staining of T lymphocyte infiltrates in 2% DSS vs CNF1+DSS- treated animals. (A) Bar plots showing the density of the indicated marker or marker combination in the epithelium or stroma compartments of colon tissue. **(B)** Representative images of colons from the indicated treatment groups (N=7 per group) multiple stained for EpCAM (white), CD4 (yellow), CD8 (orange), PD1 (light blue), Foxp3 (green) and DAPI (blue). **(C)** Boxplot of Foxp3 and PD1 expression in the stromal compartment of colon sections. Center lines show the medians; box limits indicate the 25th and 75th percentiles; whiskers represent Min and Max values. **(D)** Representative images of each single channel.
